# RNA 5‐Methylcytosine Modification Regulates Vegetative Development Associated with H3K27 Trimethylation in *Arabidopsis*

**DOI:** 10.1002/advs.202204885

**Published:** 2022-11-16

**Authors:** Daolei Zhang, Weijun Guo, Ting Wang, Yifan Wang, Liang Le, Fan Xu, Yue Wu, Hada Wuriyanghan, Zinmay Renee Sung, Li Pu

**Affiliations:** ^1^ Biotechnology Research Institute Chinese Academy of Agricultural Sciences Beijing 100081 P. R. China; ^2^ School of Life Science Inner Mongolia University Hohhot 010021 P. R. China; ^3^ Shangrao Normal University Shangrao 334001 P. R. China; ^4^ Department of Plant and Microbial Biology University of California Berkeley CA 94720 USA

**Keywords:** *Arabidopsis*, EMBRYONIC FLOWER1, histone 3 lysine27 trimethylation, RNA 5‐cytosine methylation, vegetative growth

## Abstract

Methylating RNA post‐transcriptionally is emerging as a significant mechanism of gene regulation in eukaryotes. The crosstalk between RNA methylation and histone modification is critical for chromatin state and gene expression in mammals. However, it is not well understood mechanistically in plants. Here, the authors report a genome‐wide correlation between RNA 5‐cytosine methylation (m^5^C) and histone 3 lysine27 trimethylation (H3K27me3) in *Arabidopsis*. The plant‐specific Polycomb group (PcG) protein EMBRYONIC FLOWER1 (EMF1) plays dual roles as activators or repressors. Transcriptome‐wide RNA m^5^C profiling revealed that m^5^C peaks are mostly enriched in chromatin regions that lacked H3K27me3 in both wild type and *emf1* mutants. EMF1 repressed the expression of m^5^C methyltransferase *tRNA specific methyltransferase 4B* (*TRM4B*) through H3K4me3, independent of PcG‐mediated H3K27me3 mechanism. The 5‐Cytosine methylation on targets is increased in *emf1* mutants, thereby decreased the mRNA transcripts of photosynthesis and chloroplast genes. In addition, impairing *EMF1* activity reduced H3K27me3 levels of PcG targets, such as starch genes, which are de‐repressed in *emf1* mutants. Both EMF1‐mediated promotion and repression of gene activities via m^5^C and H3K27me3 are required for normal vegetative growth. Collectively, t study reveals a previously undescribed epigenetic mechanism of RNA m^5^C modifications and histone modifications to regulate gene expression in eukaryotes.

## Graphical abstract

The plant‐specific protein EMBRYONIC FLOWER1 (EMF1) mediates a genome‐wide correlation between RNA 5‐cytosine methylation (m^5^C) and histone 3 lysine27 trimethylation (H3K27me3). EMF1 plays a dual role in regulating target gene expression as activator or repressor through EMF1‐mediated m^5^C or H3K27me3 modules. The integration of these two aspects of the dynamic epigenome is required for vigorous vegetative growth in plants.

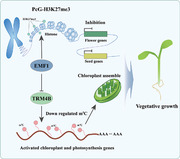

## 1 Introduction

Epigenetic modifications are important mechanisms of gene regulation.^[^ ^1^, ^2^, ^3^, ^4^ ^]^ More recently, it was discovered that mRNA undergoes similar chemical modifications, such as N6‐methyladenosine (m^6^A) and 5‐methylcytosine (m^5^C) mRNA modification, which impact transcription, RNA stability and translation, adding another layer of gene regulation.^[^ ^5^, ^6^, ^7^, ^8^ ^]^ m^6^A is the best understood and most frequent mark of mRNA with demonstrated functions ranging from pre‐mRNA processing, translation, miRNA biogenesis to mRNA decay.^[^ ^8^, ^9^ ^]^ By contrast, research on m^5^C is limited, especially in plants. Technical advances in detecting RNA modification led to the exploration of the m^5^C landscapes, their functions on RNA metabolism, as well as impact on plant development and stress responses in *Arabidopsis* and rice.^[^ ^5^, ^7^ ^]^

In addition to advances that substantiated the significance of RNA modification in controlling RNA stability, translation efficiency, and transcript levels, rapidly accumulating evidence showed significant crosstalk between RNA methylation and histone epigenetic mechanisms in mammals.^[^ ^10^, ^11^, ^12^, ^13^ ^]^ Wang et al. discovered that m^6^A regulates gene expression in neural stem cells by altering the transcript stability of proteins that deposit acetylation of histone 3 lysine27 (H3K27).^[^ ^14^ ^]^ A recent study found that bidirectional crosstalk between m^6^A and histone 3 lysine27 trimethylation (H3K27me3) acts as a critical regulatory checkpoint that controls inflammatory gene expression.^[^ ^15^ ^]^ Similar to the mode of regulation imparted by H3K27me3 and m^6^A crosstalk, a study suggests that m^6^A writer methyltransferase 3 (METTL3)/METTL14 regulates the repressive histone mark histone 3 lysine9 dimethylation (H3K9me2) and co‐transcriptionally regulates downstream genes.^[^ ^11^ ^]^ histone 3 lysine36 trimethylation (H3K36me3) as a transcription elongation marker that is enriched mainly in coding sequences (CDSs) has been shown to guide m^6^A deposition.^[^ ^10^ ^]^ In humans and mice, m^6^A directly impacts heterochromatin formation by regulating histone 3 lysine9 trimethylation (H3K9me3) levels at target sites.^[^ ^16^, ^17^, ^18^ ^]^ These studies provide direct mechanistic insights that interactions of m^6^A with histone modifications co‐transcriptionally specify transcriptional outputs, translation, recruitment of chromatin modifiers, as well as the deployment of the m^6^A methyltransferase complex (MTC) at target sites in mammals.^[^ ^10^, ^11^, ^14^, ^16^, ^19^ ^]^ However, the interplay of m^5^C RNA modification and functionally relevant histone modifications in eukaryotes, especially in plants, remains largely unknown.

Plant development progresses through distinct phases: Embryo development, seedling germination, vegetative growth, followed by a flower development and eventually seed set and senescence.^[^ ^20^, ^21^ ^]^ The transitions between these phases are controlled by distinct genetic circuits that integrate endogenous and environmental cues. For each phase, specific gene programs are activated and programs of other phases are repressed. For example, after germination, photosynthesis and chloroplast genes are activated, while seed and flower gene programs are repressed to allow for vegetative shoot development. Over the past decade, extensive studies have demonstrated epigenetic mechanisms, such as DNA methylation, RNA methylation, histone modifications, and non‐coding RNAs, as contributing factors in maintaining stable meristematic and differentiation states, and reprogramming cell fates and memory during phase changes.^[^ ^1^, ^3^, ^7^, ^22^ ^]^ However, how the different epigenetic mechanisms are integrated and coordinated in each developmental phase is so far unknown.

Polycomb group (PcG) proteins form several types of chromatin‐modifying complexes to implement repressive chromatin modifications and organization at target loci in maintaining gene silencing, including Polycomb repressive complex 1 (PRC1) and PRC2.^[^ ^23^, ^24^ ^]^ PRC2 consists of multiple proteins, EMBRYONICFLOWER 2 (EMF2), VERNALIZATION 2 (VRN2), FERTILIZATIONINDEPENDENT SEED 2 (FIS2), FERTILIZATION INDEPENDENTENDOSPERM (FIE), and MULTICOPY SUPPRESSOR OF IRA1 (MSI1), as well as the methyltransferases that trimethylate H3K27, CURLY LEAF (CLF), MEDEA (MEA), and SWINGER (SWN).^[^ ^23^, ^25^ ^]^ PRC1 includes the four proteins responsible for H2A ubiquitination, AtRING1A/1B and AtBMI1A/1B.^[^ ^26^, ^27^ ^]^ PRC2 catalyzes repressive histone H3K27me3, whereas PRC1 functions to maintain H3K27me3, deposit histone H2A monoubiquitination (H2Aub), and/or compact chromatin to inhibit transcription.^[^ ^28^ ^]^ In plants, PcG proteins are required for the transition, such as from vegetative to reproductive development, through the repression of flower organ and flowering time genes via the deposition of H3K27me3 marks.^[^ ^29^, ^30^ ^]^ EMBRYONIC FLOWER1 (EMF1) is a plant‐specific protein which has been considered a member of PRC1 owing to its Psc‐like property and interaction with *Arabidopsis* PRC1 components, AtRING/AtBMI.^[^ ^23^, ^29^ ^]^ Unlike AtRING1/AtBMI1‐harboring PRC1 has H2Aub activity,^[^ ^31^, ^32^ ^]^ the H2Aub levels did not change in *emf1‐2* mutants.^[^ ^32^, ^33^ ^]^ Instead, recent data showed that EMF1 co‐purifies with PRC2^[^ ^34^, ^35^, ^36^ ^]^ and is required for H3K27me3 marking.^[^ ^29^, ^37^, ^38^ ^]^ Nevertheless, EMF1 physically associates with components of both PRC1 and PRC2,^[^ ^29^, ^35^ ^]^ playing a vital role through H3K27me3 as well as histone 3 lysine4 trimethylation (H3K4me3) in the PcG‐mediated floral and seed developmental repression mechanism to allow vegetative growth and specify cell fates during growth and differentiation.^[^ ^24^, ^29^, ^37^, ^39^ ^]^

In plants, vigorous vegetative growth after germination requires chloroplast development and photosynthesis for subsequent energy‐intensive flowering and seed production. The *EMF1* gene can repress seed and flower gene expression via the PcG mechanism during *Arabidopsis* vegetative development. However, the epigenetic mechanism in promoting photosynthesis and chloroplast development is less clear. Here, we report the involvement of a novel epigenetic mechanism, RNA m^5^C, in EMF1 mediated‐photosynthesis gene expression, thus enabling vigorous vegetative growth. We found EMF1 acts to not only repress via PcG‐mediated H3K27me3, but also promote the expression of sets of genes during vegetative development via RNA m^5^C in *Arabidopsis*. Transcriptome‐wide profiling of RNA m^5^C reveals a genome‐wide reverse correlation between histone modification H3K27me3 and RNA methylation m^5^C. Removing *EMF1* function resulted in a global increase in m^5^C and decrease in H3K27me3 modification. m^5^C peaks were mostly enriched in regions of chromatin that lacked H3K27me3 in both WT and *emf1* mutants. EMF1 represses starch genes through PcG‐mediated repressive histone mark H3K27me3 (EMF1‐PcG‐H3K27me3), while activate expression of chloroplast and photosynthesis genes via m^5^C methyltransferase tRNA specific methyltransferase 4B (TRM4B) ‐meidated RNA methylation (EMF1‐TRM4B‐m^5^C). The opposite regulation of gene expression of diverse gene programs is both required for vigorous vegetative growth in *Arabidopsis*. Collectively, our findings revealed that EMF1 modulates *Arabidopsis* development through diverse epigenetic modifications to control gene programs needed for vegetative growth, which provide a novel epigenetic mechanism involved in histone modification and RNA m^5^C methylation and therefore adds another layer of complexity for gene regulation in eukaryotes.

## 2 Results

### 2.1 Loss of EMBRYONIC FLOWER1 Function Causes Defective Chloroplast Development, Impaired Photosynthesis, and High Starch Accumulation in *Arabidopsis*

**Figure 1:**
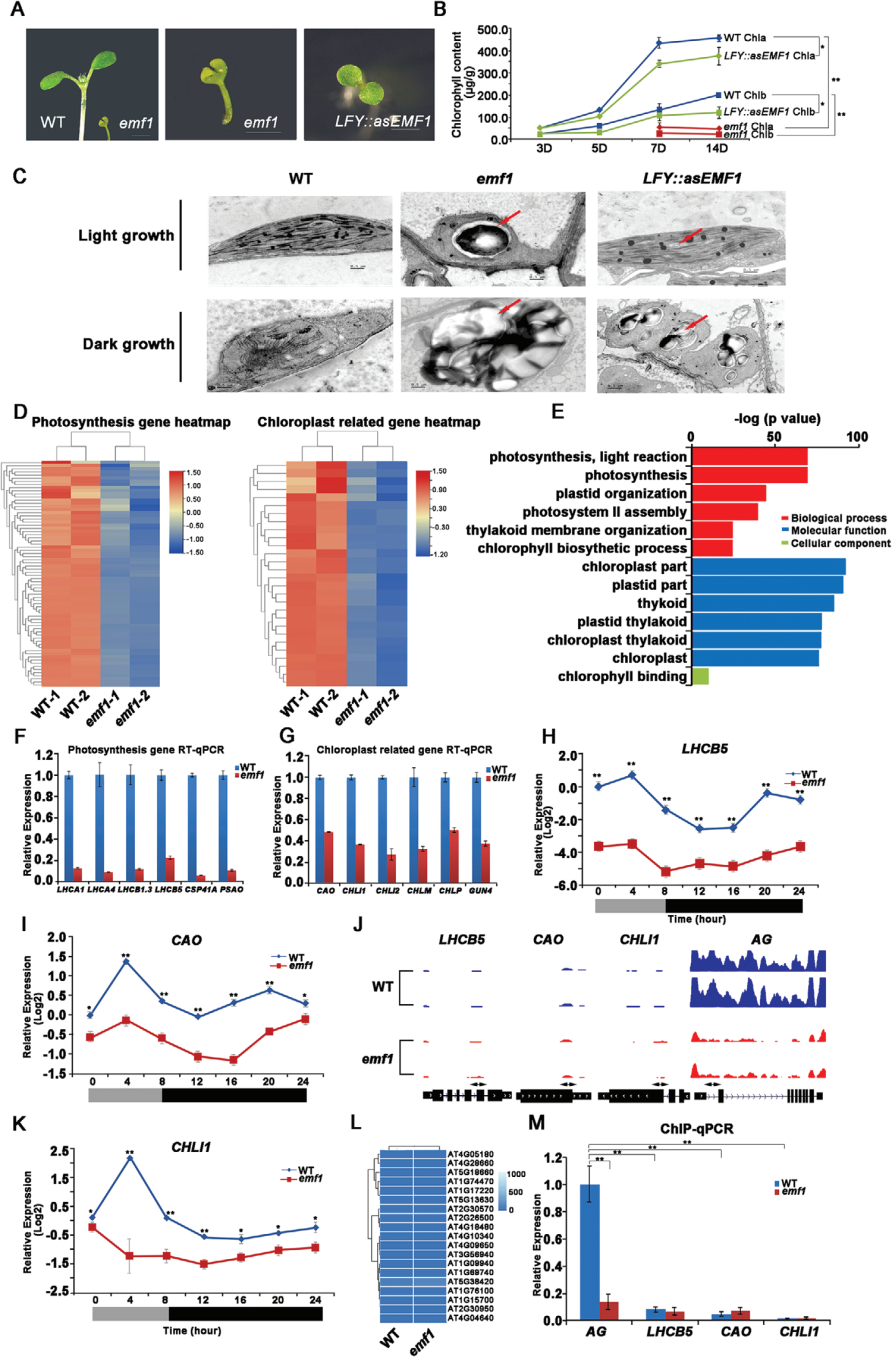
Loss of *EMF1* function causes defective chloroplast organization, impaired photosynthesis and EMF1 positively regulate gene expression of photosynthesis and chloroplast related‐genes not methylated by H3K27me3. A) Phenotypes of 7‐day‐old WT, *emf1*, and *LFY::asEMF1* plants. BAR = 1 mm. B) Chlorophyll content in 3‐day‐old, 5‐day‐old, 7‐day‐old, and 14‐day‐old of WT, *emf1*, and *LFY::asEMF1* seedlings, except for 3‐day‐old and 5‐day‐old *emf1* seedlings because *emf1/emf1* plants are not morphologically distinct from the WT/WT and WT/*emf1* plants at early seedling stage. Error bars represent ± SD (*n* = 6). C) Ultrastructure of chloroplasts of cotyledons from WT, *emf1*, and *LFY::asEMF1* under light (upper panel) and dark (bottom panel) growth condition. The red arrows indicate starch granules. BAR = 0.5 µm. D) Heat maps illustrating changes in the transcript levels of the photosynthesis genes (left panel) and chloroplast developmental genes (right panel). E) GO enrichment analysis of the downregulated genes in *emf1* mutants. F,G) Transcript levels of photosynthesis genes *LHCA1, LHCA4, LHCB1.3, LHCB5, CSP41A, PSAO*, and chloroplast developmental genes *CAO, CHLI1, CHLI2, CHLM, CHLP, GUN4* in WT and *emf1* seedlings. Graphs show the relative expression levels measured by RT‐qPCR, normalized to a *ACT8* reference gene. Error bars represent SD. H,I,K) Quantitative RT‐qPCR of photosynthesis genes and chloroplast development genes, *LHCB5*, *CAO*, and *CHLI1* over 24 h in WT (blue) and *emf1* (red). Values are the means of three independent measures. Thin vertical bars represent the standard error value. Open bar, illuminated period; closed bar, dark period. J) ChIP‐seq analysis of H3K27me3 distribution at the photosynthesis and chloroplast gene loci, *LHCB5*, *CAO*, and *CHLI1*, and the flower MADS box gene *AG* in WT and *emf1* plants. Gene models shown at the bottom include 5’ UTR (medium black line), exons (black boxes), introns (thin black line), and 3’ UTR (medium black line). The white arrow indicates transcriptional direction. L) Heatmap of H3K27me3 methylation of photosynthesis, chloroplast and flower genes in the WT and *emf1* mutants. M) ChIP‐qPCR analysis of H3K27me3 levels at the *LHCB5*, *CAO*, *CHLI1*, and *AG* gene loci in WT and *emf1* plant seedlings. Primers (double arrowheads) correspond to the gene regions shown in (J). Quantities of DNA fragments after ChIP were quantified by qPCR, and were subsequently normalized to the internal control (*ACT3*).

Loss‐of‐function *emf1* mutants flower upon germination and produced dwarf plants (**Figure** **1**A; Figure S1A, Supporting Information).^[^ ^24^, ^29^ ^]^ In addition to the developmental defects, *emf1* mutants displayed yellow to light green color compared to dark green in wild‐type (WT) plants (Figure 1A; Figure S1A, Supporting Information). Similar to the null mutant *emf1*, the transgenic *LFY::asEMF1* plants expressing *antisense‐EMF1* under the control of a *LEAFY* promoter displayed a weak phenotype by producing a small shoot with several vegetative leaves that are also light green (Figure 1A; Figure S1A, Supporting Information).^[^ ^40^, ^41^ ^]^ We then investigated the chlorophyll and carotenoid content which is commonly responsible for the leaf color,^[^ ^42^ ^]^ and found the total chlorophyll and carotenoid levels as well as the ratio of chlorophyll a/b were significantly reduced in the *LFY::asEMF1* and *emf1* mutants compared with WT plants (Figure 1B; Figure S1B, Supporting Information). We further examined the photosynthetic capacity of plants impaired in *EMF1* compared with the WT plants. The Chlorophyll fluorescence induction experiments showed that the ratio of variable fluorescence to maximum fluorescence (Fv/Fm), which reflects maximum photochemical efficiency of photosystem II (PSII), was significantly lower in *LFY::asEMF1* and *emf1* seedlings than in WT plants (Figure S1C,D, Supporting Information). This result was supported by further analysis of photosynthetic pigment‐containing complexes and their protein levels (Figure S1E,F, Supporting Information), suggesting that the plant specific PcG protein EMF1 might be involved in photosynthetic system and chloroplast development in *Arabidopsis*.

The pale‐green leaves and decreased photosynthetic efficiency in *emf1* mutants suggests defective chloroplast development. We thus examined the ultrastructure of chloroplasts in WT, *emf1* and *LFY::asEMF1* mutant seedlings via transmission electron microscopy (TEM). TEM results showed that chloroplasts in cotyledons, hypocotyls and true leaves of WT seedlings were crescent‐shaped with a well‐formed thylakoid system of stroma thylakoids and grana thylakoids, while decreased number of thylakoid lamellar layers and poor arrangement of grana were observed in both *emf1* mutants and *LFY::asEMF1* plants (Figure 1C; Figure S1G, Supporting Information). Interestingly, we found bigger and increased number of granules accumulated in *LFY::asEMF1* and *emf1* mutants, suggesting more starch accumulated in mutant seedlings (Figure 1C; Figure S1G, Supporting Information). Consistent with this, starch content was greatly increased in the *LFY::asEMF1* (2.5 times at 7 days after germination (DAG) and 1.4 times at 14 DAG) and *emf1* (7.3 times at 7 DAG and 6.9 times at 14 DAG) mutants compared with WT seedlings (Figure 3A). Unexpectedly, we noted that impaired photosynthetic capacity in *emf1* mutants did not decrease starch granules. Since light promotes carbon fixation during photosynthesis, which in turn lead to starch synthesis, we tested whether granule accumulation would occur in the dark. Strikingly, we found that severely altered chloroplasts were still filled with great granules in *LFY::asEMF1* and *emf1* mutants under darkness (Figure 1C; Figure S1G, Supporting Information). This is a potentially important finding because increased starch granules were independent of light or photosynthesis. Rather, the increased starch accumulation in mutants might result from the reproductive state of the *emf1* mutants, which allowed the constitutive expression of flower and seed genes including starch‐related genes.^[^ ^24^, ^29^ ^]^ Therefore, we further analyzed the starch content of seeds in the early developmental stage by using WT and *LFY::asEMF1* plants, and the results showed that the starch levels in the *LFY::asEMF1* plants was also increased in the early stage of seed development (Figure S3G, Supporting Information). Together, these results suggest that EMF1 promote photosynthesis and inhibit starch production to allow for vegetative development.

### 2.2 EMBRYONIC FLOWER1 Positively Regulate Expression of Photosynthesis‐ and Chloroplast‐Related Genes that are not Trimethylated on Histone 3 Lysine27

To investigate the mechanism of EMF1 regulation on photosynthesis and chloroplast development, we first analyzed published data of mRNA transcript levels in WT and *emf1* seedlings,^[^ ^24^ ^]^ and found that the mRNA levels of photosynthesis and chloroplast genes were significantly decreased when *EMF1* expression was reduced (Figure 1D; Figure S1H, Supporting Information). Gene ontology (GO) analysis showed that down‐regulated genes in *emf1* mutants mainly enriched in photosynthesis, light reaction, photosystem II assembly, chlorophyll biosynthetic process and chloroplast development (Figure 1E), which is consistent with phenotypes of defective chloroplast development, reduced chlorophyll content and low photosynthetic efficiency in *emf1* mutants (Figure 1A,B; Figure S1A‐S1F, Supporting Information). RNA‐seq results of selected photosynthesis‐ and chloroplast‐related genes were validated using RT‐qPCR (Figure 1F,G). We further analyzed dynamic expression patterns of *LIGHT HARVESTING COMPLEX OF PHOTOSYSTEM II 5* (*LHCB5*), *CHLOROPLAST SIGNAL RECOGNITION PARTICLE COMPONENT* (*CAO*), and *LOW TEMPERATURE WITH OPEN‐STOMATA 1* (*CHLI1*) genes over a day‐night cycle. Indeed, we found the transcript levels of *LHCB5*, *CAO*, and *CHLI1* in *emf1* mutants over the 24 h were much lower than those in WT plants (Figure 1H,I,K). In short, EMF1 plays a critical role in promoting the expression of photosynthesis and chloroplast genes.

EMF1 is known to be involved in PcG‐mediated transcriptional silencing through the repressive mark of H3K27me3 in plants.^[^ ^24^, ^38^, ^39^, ^41^ ^]^ To find out if photosynthesis and chloroplast genes were also marked with H3K27me3, we re‐analyzed genome‐wide ChIP‐seq data of H3K27me3 in WT and *emf1* mutants.^[^ ^24^ ^]^ We found H3K27me3 modifications on photosynthesis and chloroplast genes barely existed in either WT or *emf1* mutants by using *AGAMOUS* (*AG*) as a H3K27me3‐marked positive control (Figure 1J,L,M), indicating that positive regulation of EMF1 on these genes did not involve the H3K27 trimethylation.

### 2.3 EMBRYONIC FLOWER1 Knockout Leads to Genome‐Wide Elevation of mRNA 5‐Cytosine Methylation Modification

**Figure 2:**
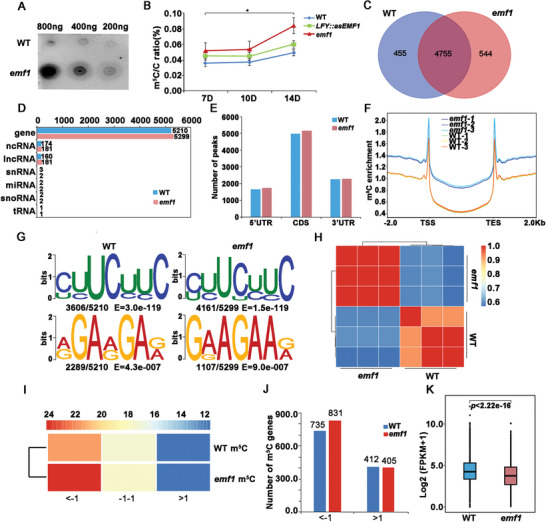
m^5^C Methylation in WT and *emf1* mutants. A) Dot blot analysis of m^5^C levels in genomic DNA purified from WT and *emf1* seedlings. B) LC‐MS/MS assays of m^5^C levels in WT and *emf1* seedlings. C) Overlap of m^5^C marked genes identified from WT and *emf1* plants by m^5^C‐RIP‐seq analysis. D) List of the numbers of m^5^C modified genes in different gene categories, gene, ncRNA, IncRNA, snRNA, miRNA, snoRNA, tRNA. E) Comparison of the number of WT m^5^C peaks versus *emf1* m^5^C peaks in transcript segments divided into 5’ UTRs, CDSs, and 3’ UTRs. F) Occupancy of m^5^C‐methylated genes around the transcription start site (TSS) and the transcription end site (TES) in WT and *emf1* plants. The m^5^C‐methylated genes expressed at WT and *emf1* mutants are plotted with different m^5^C occupancies, which represents the relative number of genes containing m^5^C sites of the total gene number in a 100‐bp window. G) Sequence logo representations of the consensus motifs identified by MEME‐ChIP in WT and *emf1* mutants. The number of occurrences of each motif relative to the total number of m^5^C peak summit and the corresponding *E* value generated by MEME‐ChIP are shown under the logo. H) Pearson correlation coefficients among gene expression profiles generated by the RNA‐seq analysis of three biological replicates. RNA‐seq was performed on RNA extracted from 7‐day‐old WT and *emf1* seedlings. High Pearson's correlation coefficients (*r* > 0.96) were obtained between all replicates, suggesting the high reproducibility of RNA‐seq data between biological replicates. I) The percentage of m^5^C‐methylated genes at a given expressed level. <‐1, down‐regulated genes in *emf1* revealed by RNA‐seq. ‐1‐1, non‐regulated genes in *emf1* revealed by RNA‐seq. >1, up‐regulated genes in *emf1* revealed by RNA‐seq. Change of expression genes in *emf1* is *p* < 0.05. The association between the number of m^5^C‐methylated genes and expression was examined by chi‐square test (*p* < 2.2e‐16). J) Comparison of number of m^5^C methylated genes in WT and *emf1* seedlings. <‐1, down‐regulated genes in *emf1* revealed by RNA‐seq. >1, up‐regulated genes in *emf1* revealed by RNA‐seq. Change of expression genes in *emf1* is *p* < 0.05. K) Box plot comparing FPKM expression levels between genes with m^5^C sites in WT and *emf1* seedlings. *p* values were calculated by performing two‐tailed unpaired Student's *t*‐test.

Since H3K27 of photosynthesis and chloroplast genes were not trimethylated, the expression of these genes cannot be regulated through the PcG mechanism. This raises the question of whether there are other epigenetic marks involved in genes positively regulated by EMF1. In *Drosophila*, a positive role for PcG proteins in transcriptional regulation occurred via H4K20me1 on genes that could be enriched by H3K27me3.^[^ ^43^ ^]^ However, the existence of H4K20 methylation has not yet been shown in plants, including *Arabidopsis*.^[^ ^44^ ^]^ Recently, m^5^C has been reported to be involved in RNA metabolism and root development in *Arabidopsis*, and photosynthesis and stress response in rice.^[^ ^5^, ^6^, ^7^ ^]^ To find out whether EMF1 is required for RNA methylation of m^5^C on *Arabidopsis* RNA, we first performed dot blot assays of the total RNA isolated from WT and *emf1* seedlings using an anti‐m^5^C antibody and found a significant elevation of m^5^C RNA modification in *emf1* mutants compared with WT control (**Figure** **2**A). Liquid chromatography coupled with tandem mass spectrometry (LC‐MS/MS) analysis confirmed that the abundance of m^5^C in RNA was elevated by 50%, 50% and 42% in *emf1* mutants at 7 DAG, 10 DAG, 14 DAG, respectively, compared with WT (Figure 2B). Similarly, m^5^C levels were also elevated in *LFY::asEMF1* plants compared with WT plants (Figure 2B; Figure S2A, Supporting Information). The LC‐MS/MS results showed that m^5^C RNA was also detected at various levels in different tissues of WT and *LFY::asEMF1* plants (Figure S2B, C, Supporting Information). The ubiquitous presence of m^5^C is similar to that of *EMF1* expression pattern in all plant organs.^[^ ^5^ ^]^ These results suggested that plant‐specific protein EMF1 is involved in the ubiquitous presence of m^5^C RNA in *Arabidopsis*.

Next, the epitranscriptomic analysis was performed to localize m^5^C sites at the transcriptome‐wide level by carrying out m^5^C‐RIP‐seq on RNA extracted from 7 DAG WT and *emf1* seedlings when *emf1* mutants have cotyledons but have not produced floral structures to avoid drastic morphological differences between WT and *emf1* plants. We performed m^5^C‐RIP‐Seq using the same WT and *emf1* seedlings with three highly reproducible biological replicates (Figure S2E, Supporting Information). In WT plants, we identified 6572 putative m^5^C peaks (*p* ≤ 0.00001, Fold change (FC) ≥ 2) that corresponded to 5210 expressed *Arabidopsis* genes with an average of 1.26 m^5^C peaks per gene (Figure S2D and Table S1, Supporting Information), which is consistent the previous report of m^5^C epitranscriptome in *Arabidopsis*.^[^ ^5^ ^]^ Comparing with m^5^C profile of published data of genome‐wide m^5^C occupancy,^[^ ^5^ ^]^ we found two epitranscriptomic data shared 92% target loci (Figure S2E, Supporting Information), validating the accuracy and robustness of our m^5^C‐RIP‐seq results. In *emf1* mutants, we identified 6778 m^5^C peaks (*p* ≤ 0.00001, Fold change (FC) ≥ 2) that corresponded to 5299 coding gene transcripts with an average of 1.28 m^5^C peaks per gene (Figure S2D and Table S1, Supporting Information). Among them, 4755 (89.7%) m^5^C RNA methylated genes overlapped in WT and *emf1* mutants (Figure 2C). Moreover, we noticed that the number of m^5^C sites didn't exhibit significant increase in *emf1* mutants compared with WT plants (Figure 2D‐2E). Further analysis showed that in total with an average of m^5^C peaks per gene, 79%, 78% of m^5^C‐containing genes had one m^5^C methylation site in WT and *emf1* mutants, respectively (Figure S2F, Supporting Information). Consistently, highly overlapped m^5^C genes were found between WT and *emf1* mutants, especially for most genes with one m^5^C methylation peak (Figure S2F, Supporting Information). These results suggested that EMF1 does not affect the sites of majority, but a small subset of m^5^C genes in *Arabidopsis*.

We then compared the distribution of m^5^C peaks along protein coding transcripts in gene architecture including 5’ untranslated regions (UTRs), CDS, and 3’ UTRs. The majority of m^5^C peaks (>55%) were located in CDSs in both WT and *emf1* mutants (Figure S2G, Supporting Information), which is consistent with previous reports.^[^ ^5^ ^]^ By plotting the distribution of m^5^C peaks across mRNA segments, we found that m^5^C was generally enriched in CDS regions with two peaks, one located immediately after start codons and the other before stop codons (Figure S2H, Supporting Information). The distribution pattern of m^5^C was not altered in *emf1* mutants (Figure S2H, Supporting Information), but the enrichment intensity of m^5^C along mRNA transcripts was dramatically increased in *emf1* mutants compared with WT plants (Figure 2F), confirming that removing *EMF1* function mainly affected the methylation enrichment in *Arabidopsis*.

To determine whether the identified m^5^C peaks shared common sequence elements, we executed an unbiased search for consensus motifs enriched in regions surrounding m^5^C peak summits (Figure 2G). The most enriched motifs of YYTCYYCH (*E* = 3.0^e‐119^) and RGARGAR (*E* = 4.3^e‐007^) in WT plants, which were present in around 69% and 44% of the methylated peak summits, respectively (Figure 2G). These motifs were also found in *emf1* mutants with YYTYYYCH (79%, *E* = 1.5^e‐119^) and GRAGAAR (21%, *E* = 9.0^e‐007^), suggesting that *EMF1* did not affect most of the m^5^C enriched motifs.

### 2.4 EMBRYONIC FLOWER1‐Dependent 5‐Cytosine Methylation Populates the Down‐Regulated Genes

In mammals and plants, m^5^C is involved in mRNA decay and translation.^[^ ^5^, ^6^, ^7^, ^45^ ^]^ However, the relationship between m^5^C levels and mRNA abundance was unclear, especially in plants, despite several recent studies with conflicted results in *Arabidopsis* and rice.^[^ ^5^, ^6^, ^7^ ^]^ To investigate the relationship between m^5^C RNA methylation and transcript abundance, we performed RNA‐Seq using the same WT and *emf1* seedlings with three highly reproducible biological replicates (Figure 2H; Figure S1I, Supporting Information). We identified 3526 up‐regulated (Log2 FC ≥ 1), 3377 down‐regulated (Log2 FC ≤ −1), as well as, 16931 no‐change (−1 < Log2 FC > 1) genes in *emf1* compared with WT seedlings (Table S2). Based on expression levels in mutants, we divided the genes into 3 groups, up‐regulated, no‐change, down‐regulated and compared the distribution of transcript abundance with m^5^C levels. In *emf1* mutants, a significant number of down‐regulated genes were methylated at m^5^C sites (831, *p* < 2.2e‐16) (Figure 2I,J). Interestingly, we found that m^5^C enrichment on up‐regulated genes was hardly changed in *emf1* mutants (Figure 2I,J), suggesting EMF1‐mediated m^5^C function specifically on down‐regulated genes. Further analysis showed that m^5^C modification was increased in *emf1* mutants on low expressed genes (FPKM < 1), while the majority of highly expressed genes (FPKM ≥ 1) showed no change in m^5^C modification in WT and *emf1* plants (Figure S2I, Supporting Information). m^5^C‐methylated genes were expressed at significantly lower levels in *emf1* mutants than in WT plants (*p* < 2.22e‐16) (Figure 2K). In *emf1* mutants, m^5^C‐methylated mRNA was more abundant in down‐regulated (876, 17%) genes than in up‐regulated genes (439, 8%) (Figure S2J, Supporting Information). Thus, the association of increased m^5^C levels with reduced mRNA abundance indicated that m^5^C mRNA modification negatively impacts transcription which is consistent with previous study in *Arabidopsis* and other species.^[^ ^6^, ^46^, ^47^ ^]^

To confirm the effect of m^5^C modification on gene expression, we examined m^5^C levels of selected m^5^C‐methylated genes (Figure S3A, B, Supporting Information), including highly m^5^C‐methylated genes such as *PHOTOSYSTEM I SUBUNIT O* (*PSAO*), *CHLOROPLAST RNA BINDING PROTEIN 33A* (*CP33*), *PHOTOSYSTEM I SUBUNIT E‐2* (*PSAE2*), and *CHLOROPLAST STEM‐LOOP BINDING PROTEIN OF 41 KDA* (*CSP41A*), and low‐level methylated genes such as *Toll‐Interleukin‐Resistance domain family protein* (*TIR*), *a subtilisin‐like serine protease* (*SBT1*), *Kratos restricts cell death during differentiation of tracheary elements* (*KRATOS*), and *MYB DOMAIN PROTEIN 77* (*MYB77*) (Figure S3A,B, Supporting Information). m^5^C‐RIP‐qPCR validated that, in *emf1* mutants, m^5^C modification increased in *PSAO*, *CP33*, *PSAE2*, and *CSP41A* mRNA, whereas decreased in *TIR*, *SBT1*, *KRATOS*, and *MYB77* (Figure S3C, E, Supporting Information). RT‐qPCR analysis verified the mRNA expression of these genes in *LFY::asEMF1* and *emf1* mutants were inversely correlated m^5^C modification (Figure S3D, F, Supporting Information).

### 2.5 EMBRYONIC FLOWER1 Maintains the Repression of Starch Synthesis Genes via Histone 3 Lysine27 Trimethylation

**Figure 3:**
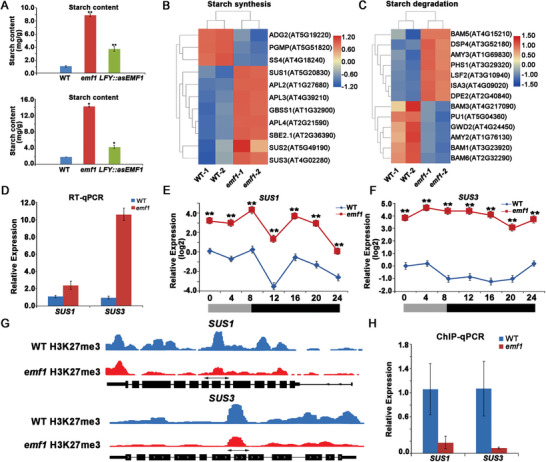
Loss of *EMF1* function causes defective highly accumulated starch in seedlings. A) Changes of starch content in WT, *emf1*, and *LFY::asEMF1* plans at 7 days after germination (DAG, top panel) and 14 DAG (bottom panel). B,C) Heat maps of RNA‐seq analysis showing differently expressed starch synthesis genes (right) and starch degradation genes (left) in *emf1* versus WT. D) mRNA expression levels of the starch synthesis genes *SUS1* and *SUS3* in WT and *emf1* seedlings. Graphs show the relative expression levels measured by Quantitative RT‐qPCR, normalized to a *ACT8* reference gene. Error bars represent SD. E,F) Quantitative RT‐qPCR of *SUS1* and *SUS3* over 24 h in WT (blue) and *emf1* (red). Values are the means of three independent measures. Thin vertical bars represent the standard error value. Open bar, illuminated period; closed bar, dark period. G) ChIP‐seq analysis of H3K27me3 distribution at *SUS1* and *SUS3* loci in WT, *emf1* seedlings. Gene models shown at the bottom include 5’ UTR (medium black line), exons (black boxes), introns (thin black line), and 3’ UTR (medium black line). H) ChIP‐qPCR analysis of H3K27me3 levels at the *SUS1* and *SUS3* loci in WT and *emf1* seedlings. Primers (double arrowheads) correspond to the gene regions shown in (G). Quantities of DNA fragments after ChIP were quantified by qPCR, and were subsequently normalized to the internal control (*ACT3*). The fold changes of *SUS1* and *SUS3* are shown.

We have found starch over‐accumulation in *emf1* seedlings. To investigate how *EMF1* affects starch accumulation, we analyzed the transcriptional profiles of starch‐related genes including starch biosynthesis and degradation genes.^[^ ^24^ ^]^ We found that most starch synthesis genes (73%) are up‐regulated in *emf1* mutants (**Figure** **3**B), while starch degradation genes are evenly up‐ or down‐regulated (Figure 3C). Thus, the highly ectopically expressed starch synthesis genes were likely to be responsible for starch accumulation in *emf1* mutants. It has been reported that S*ucrose synthase 1 (SUS1)* and *SUS3* were important for starch synthesis in *Arabidopsis* and other plant species.^[^ ^48^, ^49^, ^50^, ^51^, ^52^, ^53^ ^]^ Therefore, we chose these two genes for further analysis. We first confirmed that *SUS1* and *SUS3* were up‐regulated in *emf1* via RT‐qPCR (Figure 3D). We then investigated dynamic expression profiles of *SUS1* and *SUS3* in WT and *emf1* plants over 24 h, and found their diurnal expression patterns in WT plants were significantly increased in *emf1* mutants (Figure 3E,F). Thus, the starch granules accumulation in *emf1* seedlings is likely caused by the enhanced starch synthesis in the absence of EMF1‐mediated transcriptional repression. Since *EMF1* is involved in PcG‐mediated gene silencing through H3k27 trimethylation,^[^ ^24^ ^]^ we analyzed the H3K27me3 modification in WT and *emf1* mutants by using previous ChIP‐seq data.^[^ ^24^ ^]^ Indeed, we found that the H3K27me3 enrichment was greatly reduced on *SUS1* and *SUS3* genes in *emf1* mutants compared WT plants (Figure 3G), which was further confirmed by ChIP‐qPCR analysis (Figure 3H), consistent with their increased transcriptional levels. Taken together, these results confirmed that EMF1 indeed maintain repressive state of expression on starch biosynthesis genes via the PcG mechanism.

### 2.6 Inverse Association of 5‐Cytosine Methylation RNA Modification with Histone 3 Lysine27 Trimethylation

**Figure 4:**
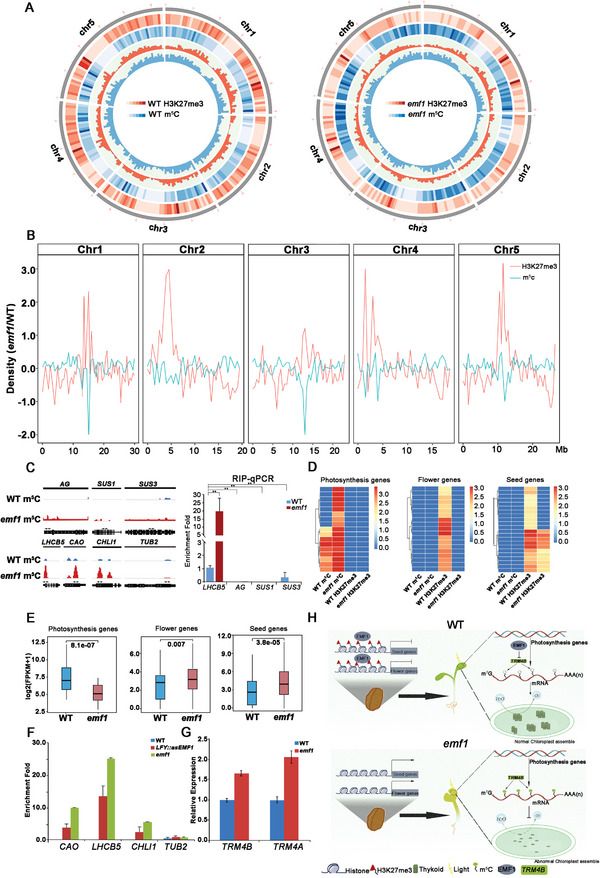
EMF1 is required for m^5^C RNA and H3K27me3 modifications which affect chromatin state and downstream transcription. A) Circos plots of the m^5^C methylation in WT and *emf1* seedlings, respectively. Four rings from outside to inside show genomic positions (1st), H3K27me3 enrichment fold (profiled by ChIP‐seq) (2nd), m^5^C enrichment fold (profiled by RIP‐seq) (3rd), H3K27me3 peak density (4th), m^5^C peak density (5th). B) Distribution of m^5^C (blue) and H3K27me3 (red) along the five *Arabidopsis* chromosomes. C) m^5^C distribution at mRNA of flower gene *AG*, starch synthetic genes *SUS1* and *SUS3*, photosynthetic and chloroplast genes *LHCB5*, *CAO*, *CHLI1*, and negative control gene *TUB2* (left). RIP‐qPCR analysis of m^5^C levels at the *AG*, *SUS1*, and *SUS3* gene loci in WT and *emf1* plant seedlings by using *LHCB5* as a positive control of m^5^C‐marked gene. Primers (double arrowheads) correspond to the gene regions shown in (C). Quantities of RNA fragments after RIP were quantified by qPCR, and were subsequently normalized to the internal control (*TUB2*). D) Heat map showing methylation of EMF1‐dependent m^5^C sites and H3K27me3 sites in WT and *emf1* (*p* ≤ 0.0001). E) Box plot comparing FPKM expression levels among photosynthesis, flower and seed genes marked with m^5^C methylation in WT and *emf1* seedlings. *p* values were calculated by two‐tailed unpaired Student's t‐test. F) m^5^C‐IP‐qPCR analysis of m^5^C modification on mRNA of *LHCB5*, *CAO*, *CHLI1* in *LFY::asEMF1* and *emf1* plants. Error bars, mean ± SD, *n* = 3. G) Quantitative RT‐qPCR analyses of expression of m^5^C methyltransferase genes, *TRM4A* and *TRM4B* in WT and *emf1* seedlings. Gene expression levels in WT seedlings are set as 1. Error bars, mean ± SD, *n* = 3. H) Working model of EMF1‐mediated m^5^C and H3K27me3 in *Arabidopsis* seedling development.

Rapidly accumulating evidence suggests that the most prevalent RNA modification, m^6^A, shows significant crosstalk with histone modifications of H3K27ac, H3K27me3, H3K9me2, and H3K36me3 to regulate chromatin accessibility and downstream transcription in mammals.^[^ ^13^ ^]^ Since EMF1 mediates both m^5^C and H3K27me3, we explored whether m^5^C and H3K27me3 modifications interact on the epigenome. We profiled the global m^5^C sites and H3K27me3 sites in each 50‐kb bins of the *Arabidopsis* genome with the five chromosomes occupied with different methylation levels (**Figure** **4**A). Both m^5^C and H3K27me3 marks were widely distributed in all 5 chromosomes in WT and *emf1* mutants (Figure 4A). In *emf1* mutants, the enrichment of H3K27me3 modification decreased in all chromosomes, while m^5^C increased in all chromosomes compared to WT plants (Figure 4A). Comparing the global distribution of m^5^C and H3K27me3 modification sites, we found that m^5^C peaks were mostly enriched in chromatin regions in which H3K27me3 was sparse, and the reverse is also true (Figure 4B). In agreement with this, the distribution of H3K27me3 exhibited a strong correlation with m^5^C (*R* = −0.42, *p* = 1.1^e−11^) (Figure S3H, Supporting Information). In addition, transcripts produced from chromatin without H3K27me3 were highly enriched for m^5^C compared to transcripts from H3K27me3 marked chromatin (Figure S3I, Supporting Information). Therefore, these results suggested that an exclusion of m^5^C from H3K27me3‐makred chromatin in *Arabidopsis*. In addition, we compared the global distribution of m^5^C with other epigenetic modifications, including N6‐methyldeoxyadenosine (6mA) 5‐methylcytosine (5mC), H3K4me3, and H2AK121ub modifications, but these modifications didn't exhibit a complementary distribution pattern with m^5^C in *Arabidopsis* genome (Figure S4A–D, Supporting Information).

Next, we examined the interplay of RNA m^5^C methylation and H3K27me3 on target gene regulation. We found that mRNA of non‐H3K27me3 marked genes, such as photosynthesis and chloroplast genes, *LHCB5*, *CAO*, and *CHLI1*, were marked with m^5^C and their mRNA was notably decreased in *emf1* mutants (Figure 4C; Figure S6A, Supporting Information). While H3K27me3 marked genes, such as flower genes and starch synthetic genes, *AG*, *SUS1*, and *SUS3*, were non‐m^5^C modified genes by using *LHCB5* as a m^5^C positive control (Figure 4C). As shown in Figure 4D, the RNA of photosynthesis, flower and seed genes displayed opposite and complementary distribution of m^5^C and H3K27me3 modifications, respectively (Figure 4D). Thus, m^5^C‐methylated mRNA of photosynthesis genes accumulated at significantly lower levels in *emf1* mutants (*p* = 5.1^e‐07^), while H3K27me3‐methylated flower genes (*p* = 7.0^e‐03^) and seed genes (*p* = 3.8^e‐05^) were expressed at significantly higher levels in mutants (Figure 4E). These results indicated that EMF1‐mediated H3K27me3 and m^5^C RNA modification play a mutually exclusive role in regulating diverse sets of genes.

### 2.7 EMBRYONIC FLOWER1 Deficiency Causes de Novo 5‐Cytosine Methylation and Low Transcripts of Photosynthesis and Chloroplast Genes

Although our results showed that EMF1 does not affect the sites of majority of m^5^C genes (89.7%), there is a small subset of novel m^5^C sites identified in *emf1* mutants (Figure 2C). Since m^5^C RIP‐seq revealed a global hypermethylation of m^5^C in the transcriptome upon EMF1 depletion, we then set out to identify EMF1's m^5^C RNA target genes and observed that 1895 m^5^C‐marked genes displayed a dramatic increase or decrease in m^5^C levels in *emf1* mutants compared with WT plants (Figure S5A, Supporting Information). Most of m^5^C‐methylated genes (1726/1895, 91%) showed significantly elevated m^5^C levels (Figure 2F; Figure S5A, Supporting Information). Notably, 544 out of the 1726 (32%) hypermethylated m^5^C‐methylated genes were de novo m^5^C methylation which partially contributed to greater m^5^C patterns in *emf1* mutants (Figure S5A and Table S3, Supporting Information). Integrated analysis of m^5^C RIP‐seq and RNA‐seq data revealed genes with increased m^5^C levels tend to be down‐regulated in *emf1* mutants with a negative correlation (*R* = −0.28, *p* = 2.0^e‐3^) between the m^5^C levels and mRNA abundance (Figure S5B, Supporting Information), further demonstrating that m^5^C modifications are associated with low transcription activity.

To investigate the function of EMF1‐mediated m^5^C RNA modification, we performed GO analysis for genes with no‐H3K27me3 methylation, increased m^5^C, de novo m^5^C, and decreased m^5^C levels in *emf1* mutants. The results revealed distinct functional groups in which no‐H3K27me3 methylation genes were mainly clustered in photosynthesis and chloroplast related genes (Figure S5C, Supporting Information), increased m^5^C‐methylated genes were mainly involved in negative regulation of multiple biological processes and chloroplast development (Figure S5D, Supporting Information), and the genes with de novo m^5^C were mainly clustered in photosynthesis (Figure S5E, Supporting Information). We also found that the small group of genes with decreased m^5^C was mainly involved in biological processes, including plant response stresses, stimulus, and root development (Figure S5F, Supporting Information), which is consistent with the previous report about m^5^C in response to stress and root development in plants.^[^ ^5^, ^6^ ^]^ We then selected three photosynthesis and chloroplast genes, *CAO*, *LHCB5*, and *CHLI1*,^[^ ^54^, ^55^, ^56^, ^57^ ^]^ to validate the enrichments of m^5^C and mRNA abundance using RIP‐qPCR and RT‐qPCR analysis. The results showed increased m^5^C levels and decreased expression of these genes in WT, *LFY::asEMF1* and *emf1* plants (Figure 4F; Figure S6A, Supporting Information). These findings confirmed that loss of EMF1 specifically decreased the expression of photosynthesis and chloroplast‐related genes during seedling development by increasing m^5^C methylation at their mRNA.

### 2.8 EMBRYONIC FLOWER1 Regulates Transcription of 5‐Cytosine Methylation Methyltransferase TRM4B through H3K4me3, Independent of Polycomb Group‐Mediated Histone 3 Lysine27 Trimethylation Mechanism

Methylation of 5‐Cytosine on mRNA occurs via methyltransferase, tRNA specific methyltransferase 4 (TRM4) or NOP2/Sun domain protein 2 (NSUN2) known as “writers.”^[^ ^5^ ^]^ To understand how EMF1 is involved in m^5^C modification, we examined whether two *Arabidopsis* methyltransferases, *TRM4A* and *TRM4B*, are regulated by EMF1. The expression of *TRM4A* and *TRM4B* were increased in *emf1* mutants, which is consistent with a marked increase of m^5^C in *emf1* mutants (Figure 4G). Since *TRM4B* has been identified as m^5^C methyltransferase in *Arabidopsis*, we compared TRM4B‐mediated m^5^C‐methylated genes^[^ ^5^ ^]^ with expression of altered genes in *emf1* mutants. We found 371 TRM4B‐mediated genes were expressed differently in *emf1* mutants, of which 249 (67%) were down‐regulated in *emf1* mutants (Figure S6B, C, Supporting Information), indicating that m^5^C mRNA modification tends to be negatively correlated with gene expression. GO analysis of TRM4B‐mediated m^5^C and down‐regulated genes in *emf1* mutants showed these genes also mainly associated with photosynthesis, chloroplast and plastids development (Figure S6D, Supporting Information). Moreover, among 483 up‐regulated genes with reduced m^5^C levels in *trm4b* mutants, 100 of them showed differential expression patterns in *emf1* mutants, in which 70% of genes were down‐regulated in *emf1* mutants (Figure S6E, Supporting Information). We further investigated whether *trm4b* mutants exhibited photosynthesis or chloroplast related phenotypes by measurement of the chlorophyll content in WT and *trm4b* mutants. Although *trm4b* mutants did not show obvious morphological phenotypes in aerial tissues as previously reported (Figure S6F, Supporting Information),^[^ ^5^ ^]^ we observed the increased chlorophyll levels in *trm4b* mutants (Figure S6F, Supporting Information). We then performed GO analysis of TRM4B‐mediated m^5^C genes with up‐regulated expression levels in *trm4b* mutants,^[^ ^5^ ^]^ and found these genes were also associated with chloroplast organization and chloroplast rRNA processing (Figure S6G, Supporting Information). RT‐qPCR analysis verified increased mRNA levels of randomly selected photosynthesis‐ and chloroplast‐related genes in *trm4b* seedlings, such as *malate dehydrogenase (MDH)*, *pentatricopeptide repeat‐containing protein (SVR7), translocon at the inner envelope membrane of chloroplasts 55‐II (TIC55), PSAO, phytochrome C (PHYC)* (Figure S6H, Supporting Information). These results are consistent with our findings that EMF1 negatively regulates m^5^C methyltransferase TRM4B to mediate m^5^C modification of photosynthesis and chloroplast genes in *Arabidopsis*.

These findings drive us to explore how EMF1 regulates *TRM4B* expression. Previous studies have shown that reducing EMF1 activity decreased H3K27me3 levels and/or increased H3K4me3 levels on target gene loci.^[^ ^24^, ^41^ ^]^ We first performed ChIP‐qPCR to analyze the H3K27me3 enrichment on TRM4B locus in WT and *emf1* mutants and found that *TRM4B* is not a H3K27me3‐modified gene (Figure S6I‐J, Supporting Information), suggesting EMF1 may regulate it through H3K4me3 epigenetic modifications. In eukaryotes, H3K4me3 is associated with active chromatin and gene expression.^[^ ^58^, ^59^ ^]^ Thus, we performed ChIP‐qPCR to analyze the H3K4me3 enrichment on *TRM4B* locus in WT and *emf1* mutants by using *AG* as a positive control of H3K4me3‐marked genes. We found that the elevated expression level of *TRM4B* was indeed associated with more enriched H3K4me3 active marks on its locus in *emf1* mutants than in WT (Figure 4G; Figure S6M, Supporting Information). These results suggest that EMF1 regulates transcription of *TRM4B* through H3K4me3, independent of PcG‐mediated H3K27me3 mechanism, and elevated expression of m^5^C methyltransferase which then facilitated m^5^C modifications in *emf1* mutants.

### 2.9 EMBRYONIC FLOWER1 Influences 5‐Cytosine Methylation Deposition in mRNA

We have shown that removing of EMF1 function results in elevated level of *TRM4B* that would increase m^5^C deposition at target genes (Figure 4G). EMF1 was found to directly bind and activate the expression of photosynthesis and chloroplast genes (Figure 1D–G; Figure S7E, Supporting Information), and TRM4B was also reported to bind target loci to promote m^5^C enrichment.^[^ ^5^, ^6^ ^]^ Thus, EMF1 might bind the same sites as TRM4B did on target loci to mediate m^5^C deposition in mRNA in *Arabidopsis*. To test this hypothesis, we first compared binding distribution patterns of EMF1‐bound and TRM4B‐dependent m^5^C sites. Previous study identified 5533 EMF1‐bound targets that were divided into two groups,^[^ ^37^ ^]^ of which 3230 highly trimethylated genes (EMF1‐K27 genes), such as flower and seed genes, and 2219 EMF1‐bound but not trimethylated genes (EMF1‐no‐K27 genes), such as photosynthesis and chloroplast genes. Among the 2219 EMF1‐no‐K27genes, RNAs of 775 genes are m^5^C marked (Figure S7A, Supporting Information). More importantly, we found that 458 out of 775 (59.1%) genes are TRM4B‐dependent m^5^C sites (Figure S7A, Supporting Information). Given EMF1 binds the same sites as TRM4B, the distribution pattern of EMF1‐no‐K27 should be matched with TRM4B‐mediated m^5^C sites. This was indeed the case because by comparing the distribution of EMF1‐no‐K27 to TRM4B‐dependent m^5^C sites,^[^ ^5^, ^37^ ^]^ we found that most of m^5^C peaks overlapped with the EMF1‐no‐K27 binding sites (*R* = 0.57, *p* < 2.2^e‐16^) (Figure S7B‐D, Supporting Information). In addition, we confirmed that TRM4B indeed bind the same target loci as EMF1 did on target loci, such as *PHOTOSYSTEM II SUBUNIT Q‐2* (*PSBQ‐2*), *ATP SYNTHASE DELTA‐SUBUNIT GENE* (*ATPD1*), *PLASTOCYANIN 1* (*PETE1*), *ALBINO 3* (*ALB3*), *the gamma subunit of Arabidopsis chloroplast ATP synthase* (*ATPC1*) (Figure S7E‐F, Supporting Information). To verify the function of TRM4B on m^5^C enrichment on target loci, we performed m^5^C RIP‐qPCR on photosynthesis‐ and chloroplast‐related genes in WT and *trm4b* mutants. The results showed that loss of *TRM4B* significantly decreased m^5^C modification levels on photosynthesis‐ and chloroplast‐related genes (Figure S6N‐O, Supporting Information), which is consistent with the previous report that TRM4B acts as a m^5^C methyltransferase in *Arabidopsi*s.^[^ ^5^ ^]^ These results further confirmed that the increased m^5^C levels were caused by the up‐regulated expression of *TRM4B* in *emf1* mutants. Taken together, in addition to its repressive action via PcG mechanism, EMF1 exerts a positive effect on the expression of photosynthesis and chloroplast genes that promote vegetative development via its modulation of *TRM4B* activity in *Arabidopsis*.

## 3 Discussion

In recent years, accumulating evidence has substantiated the significance of RNA modification such as m^6^A and m^5^C in controlling gene expression, RNA stability dynamics and translation efficiency.^[^ ^5^, ^8^ ^]^ While not the initial focus of RNA modification studies, the crosstalk between RNA methylation and other epigenetic regulatory players has received well‐deserved attention which inspired several intriguing questions moving forward. In mammals, it has been reported that m^6^A methylation may crosstalk with other chromatin modifications and these interactions specify transcriptional outputs, translation, recruitment of chromatin modifiers, as well as the deployment of the m^6^A MTC at target sites.^[^ ^13^ ^]^ H3K36me3 guides the binding of the m^6^A MTC to elongating transcripts and methylates nascent RNA co‐transcriptionally.^[^ ^10^ ^]^ m^6^A co‐transcriptionally directs KDM3B chromatin targeting through the m^6^A reader YTHDC1, leading to demethylation of H3K9me2 and ultimately promoting gene expression.^[^ ^11^ ^]^ However, all cross talks identified in mammals involved m^6^A, how m^5^C interacts with other chromatin modification remains elusive. Here, we revealed a previously undescribed correlation between RNA m^5^C and H3K27me3 modification to regulate vegetative development in plants. Our findings demonstrate an integration of RNA and chromatin modification to specify developmental gene programs.

In this study, we found a genome‐wide inverse relationship between m^5^C RNA and H3K27me3 in the control of chromatin state and gene expression. Genome‐wide m^5^C‐seq analyses showed *EMF1* mutation led to a global increase in m^5^C modification on mRNA compared with WT control in seedlings (Figure 2F). Comparison of genome‐wide distributions of H3K27me3 and m^5^C modifications showed that m^5^C were mostly in the regions of non‐H3K27me3 chromatin (Figure 4B). Transcripts of genes whose m^5^C modifications were significantly elevated were decreased in *emf1* mutants (Figure 2I–K). Loss of EMF1 function decreased global H3K27me3 levels through conventional PcG‐mediated repression and thus caused high expression of flower, seed and starch genes. Meanwhile, EMF1 represses the expression of m^5^C methyltransferase TRM4B, independent of the PcG mechanism (Figure 4G; Figure S6I‐J, Supporting Information), which consequently elevated enrichments of m^5^C and reduced transcript levels (Figure 2F,K). Hence, *emf1* mutations elevated the expression of m^5^C methyltransferase *TRM4B* and promotes m^5^C enrichment, which negatively impacted photosynthesis and chloroplast gene transcription, indicating the importance of m^5^C in vegetative development. For the exclusive occupation of H3K27me3 and m^5^C, the presence of large PcG protein complex might interfere with TRM4B, which is also a large protein complex, access to the cytosine on mRNA, and vice versa. This is similar to mammals^[^ ^15^ ^]^ that m^5^C modification may be taking place in the same space where PcG methylates H3K27. Thus, our findings uncovered the simultaneous modulation of m^5^C as well as H3K27me3 modifications mediated by EMF1 to regulate vegetative development in *Arabidopsis*.

As an epigenetic regulator, how EMF1 regulates *TRM4B* expression in *Arabidopsis*. We found that *TRM4B* is not H3K27me3‐modified gene (Figure S6I‐J, Supporting Information). Instead, EMF1 might regulate it through other epigenetic modifications, such as, H3K4me3, a well‐documented marker for active genes.^[^ ^58^, ^59^ ^]^ The trithorax group (TrxG) is an epigenetic protein complex able to regulate transcriptional activation through trimethylation of H3K4.^[^ ^23^, ^60^, ^61^ ^]^ The plant trxG factor ARABIDOPSIS HOMOLOG OF TRITHORAX1 (ATX1) has H3K4me3 methyltransferase activity^[^ ^62^, ^63^ ^]^ and promotes transcription initiation.^[^ ^64^ ^]^ The SAND domain‐containing trxG factor ULTRAPETALA1 (ULT1) interact with ATX1 physically and regulate common key target genes through H3K4me3.^[^ ^65^ ^]^ Our previous study demonstrated that EMF1 could physically interact with ULT1 associated with ATX1 to affect H3K4me3 on target loci.^[^ ^24^ ^]^ The *emf1* mutation reduced the H3K4me3 marks on flower and seed genes in *Arabidopsis*.^[^ ^24^, ^41^ ^]^ Another study reveals that EMF1 could directly interact with H3K4me3 demethylases, JMJ14, JMJ15 and JMJ18, to regulate H3K4me3 at its target loci in *Arabidopsis*.^[^ ^39^ ^]^ These results suggested that EMF1 directly regulate gene expression through modulation of H3K4me3 activation mark at target loci. Indeed, we found that *TRM4B* locus was more enriched for the H3K4me3 modification in *emf1* mutants (Figure S6M, Supporting Information), in which *TRM4B* expression was elevated (Figure 4G). In addition, we also found that trxG gene *ULT1* which promotes H3K4me3 deposition at target loci and activate gene expression was highly expressed in *LFY::asEMF1* and *emf1* mutants compared with WT plants (Figure S6K, Supporting Information). Our previous ULT1 ChIP‐seq data showed *TRM4B* is bound by ULT1.^[^ ^24^ ^]^ Then we conducted the ChIP‐qPCR analysis to test the binding of ULT1 on *TRM4B* loci in *35S::ULT1‐HA* plants and found that ULT1 can directly bind *TRM4B* (Figure S6L, Supporting Information). These results strongly suggest that EMF1 regulates TRM4B expression through H3K4me3 modification, thereby mediating RNA m^5^C modification and modulating photosynthesis and chloroplast development during vegetative growth in *Arabidopsis*. Although H3K4me3 is strongly associated with active chromatin, the debate continues whether H3K4me3 is the cause or consequence of transcriptional change.^[^ ^66^, ^67^, ^68^ ^]^ It is also possible that EMF1 may repress the expression of a yet unidentified transcription factor (TF) of the *TRM4B* gene. Thus, loss of *EMF1* function causes up‐regulation of the TF, which would activate *TRM4B* expression. Future studies need to be investigated to identify specific TFs for *TRM4B* regulation in *Arabidopsis*. In sum, our data indicates that besides global increased m^5^C levels, EMF1 may function as modulators of m^5^C methyltransferase *TRM4B* expression via affecting H3K4me3 deposition.

In mammals, m^6^A feeds back on chromatin modifiers and histones,^[^ ^11^ ^]^ the reverse is also true.^[^ ^10^, ^15^ ^]^ The demethylase KDM6B acts as guide for m^6^A installing machinery.^[^ ^15^ ^]^ H3K36me3 guides the binding of the m^6^A MTC to elongating transcripts and methylates nascent RNA co‐transcriptionally.^[^ ^10^ ^]^ How does EMF1 affect m^5^C deposition in mRNA? Consistent with the model that histone modification factors direct m^6^A deposition in mammals, we indeed found that EMF1 binds the same sites as TRM4B did on target loci in *Arabidopsis* because about 60% EMF1‐no‐K27 genes are TRM4B‐dependent m^5^C sites and most of m^5^C peaks overlapped with the EMF1‐no‐K27 binding sites (Figure S7, Supporting Information). In *emf1* mutants, the elevated TRM4B increased m^5^C deposition at target genes’ RNA, such as photosynthesis genes (Figure 4G). In addition, we further demonstrated that TRM4B and EMF1 indeed bind the photosynthesis and chloroplast gene loci, and affect their methylation levels and gene transcription (Figure 1D–E; Figure 4C–G; Figure S7D‐F, Supporting Information). Thus, it is thought that EMF1 may be crucial for the co‐transcriptional recruitment of m^5^C deposition in *Arabidopsis*. Collectively, these results support our prediction that EMF1 may play critical roles in influencing m^5^C deposition in mRNA. Although it is still emerging how precisely non‐H3K27me3 marked sites are selected for proper deposition or removal of m^5^C modifications, complex interactions between m^5^C and histone modifications are even more prevalent than previously understood, especially undescribed in plants.

In summary, we demonstrated that the plant specific PcG protein EMF1, play dual roles, depending on a particular context, acting as an activator or repressor. By repressing m^5^C methyltransferase TRM4B expression, EMF1 limits m^5^C deposition and thereby activate the mRNA transcripts of photosynthesis (EMF1‐TRM4B‐m^5^C module) (Figure 4H). On the other hand, EMF1 represses flowering, seed and starch genes via the PcG‐mediated H3K27me3 (EMF1‐PcG‐H3K27me3 module) (Figure 4H). Both gene activation and repression are necessary for vegetative development. Our findings uncover a new layer of gene expression regulation involving interplay between histone modification and RNA m^5^C methylation in plants.

## 4 Experimental Section

### Plant Materials and Growth Conditions

All materials used in this study are in the *Arabidopsis* Columbia (Col‐0) background (WT). Seeds of *LFY::asEMF1*,^[^ ^40^, ^41^ ^]^ *emf1*, *35S::ULT1‐HA* and *pEMF1::3FLAG‐EMF1* were from the lab as described previously.^[^ ^24^, ^37^ ^]^ Seeds of *trm4b* (SALK_01 4194) and *pTRM4B:TRM4B‐3HA* were kindly provided by Prof. Xiaofeng Gu, Chinese Academy of Agricultural Sciences. Seedlings were grown on half‐strength Linsmaier and Skoog media in LED chambers at 21 °C in short day conditions (8 h day/16 h night).

### Chlorophyll, Carotenoid, and Protein Contents

The chlorophyll content from isolated thylakoid membranes and chlorophyll per leaf area was determined as described previously.^[^ ^69^ ^]^ Carotenoid contents were described previously.^[^ ^70^ ^]^ The protein content from thylakoid membranes and the soluble fraction was measured as described previously.^[^ ^71^ ^]^

### Chlorophyll Fluorescence and Detection of Proteins

The seedlings were adapted under darkness for 20 min and then imaged, chlorophyll fluorescence Fv/Fm measured from intact leaves with a Plant Efficiency Analyzer (Hansatech Instruments, http://www.hansatech‐instruments.com). Thylakoid, soluble and total proteins were extracted as described in the previous study.^[^ ^72^ ^]^ Blue native (BN)‐PAGE was performed as described previously.^[^ ^69^ ^]^ BN gels were further immunoblotted, or gel strips were solubilized in Laemmli‐buffer (5% *β*‐mercaptoethanol), and thereafter the subunits of the protein complexes were separated in SDS‐PAGE and electroblotted.

### Sample Processing, Microscopy, and Starch Content

WT and mutant plants were collected for TEM processing. The samples were fixed in 2.5% glutaraldehyde in phosphate buffer (pH 7.2) for 4 h at 0 °C and further rinsed and post‐fixed overnight at 4 °C in 1% OsO_4_. Then, the samples were dehydrated in an ethanol series, infiltrated with a graded series of epoxy resin in epoxy propane, and embedded in Epon 812 resin. Thin sections were stained in uranium acetate followed by lead citrate and viewed with a transmission electron microscope. A Solarbio Detection Kit (Beijing, China) was used to determine starch content. The starch contents were detected with methods presented.^[^ ^73^ ^]^

### RNA Extraction, Library Preparation and RNA‐seq Data Analysis, and Validation

The seedlings were harvested for total RNA extraction. Total RNA was extracted using the RNeasy Plant Mini Kit (Qiagen). cDNA library preparation and paired single read sequencing were performed as described in the previous study.^[^ ^24^ ^]^ After removing adapter sequences and low‐quality reads (quality < 20), the clean reads were aligned to TAIR10 genome assembly (https://www.arabidopsis.org/) using TopHat2,^[^ ^24^ ^]^ and gene expression levels were calculated by featureCounts (https://subread.sourceforge.net/featureCounts) using default parameters. Differential expression analysis of WT and *emf1* was performed using DESeq in R software. Genes with fold change ≥2 and *p* value < 0.05 were assigned as differentially expressed genes. For real‐time quantitative PCR (RT‐qPCR) analysis, the first‐strand cDNAs were mixed with Power SYBR Green PCR Master Mix (Invitrogen) in a final volume of 20 µL. The RT‐qPCR reaction was carried out in an Applied Biosystems. All gene‐specific primers are listed in Table S4.

### Dot Blot Analysis of 5‐Cytosine Methylation Levels

Dot blot analysis was performed as previously described^[^ ^5^ ^]^ with slight modifications. The purified RNA was heated at 95 °C for 3 min, followed by chilling on ice immediately. RNA was spotted on a Hybond‐N+ membrane (Amersham), followed UV crosslinking. The membrane was washed by 1x PBST buffer (PBS with Tween‐20), blocked with 5% of non‐fat milk in PBST, and incubated with anti‐m^5^C antibody (1:1000; MAb‐081‐010, Diagenode) overnight at 4 °C. After the membrane was washed by 1x PBST, incubating with horseradiash‐peroxidase‐conjugated anti‐mouse IgG secondary antibody (Santa Cruz). The ECL Western Blotting Detection Kit (Thermo) was used to visualize the membrane.

### Liquid Chromatography Coupled with Tandem Mass Spectrometry Analysis 5‐Cytosine Methylation/C Ratio

Total RNA was isolated according to standard procedures as previous described methods.^[^ ^5^ ^]^ Samples were subjected to LC‐MS/MS analysis on an Agilent 6490 Triple Quadrupole mass spectrometer. Ribonucleosides were quantified using the nucleoside‐to‐base ion mass transitions of 258.1 to 126.1 for m^5^C, 244 to 112 for C, and 274.0 to 142.1 for hm^5^C.

### m5C‐RIP‐seq, Library Preparation, and Sequencing

m^5^C‐RIP‐seq was performed as previously described with m^5^C modifications.^[^ ^5^ ^]^ Briefly, 100 nucleotide (nt)‐long fragmented RNA was incubated with anti‐m^5^C antibody (Diagenode) and then subjected to Dynabeads protein G (Invitrogen) binding. After extensive washing, bound RNA was eluted from the beads with 6.7 mm m^5^C (Sigma‐Aldrich) in IP buffer, and precipitated by ethanol. NEBNext Ultra Directional RNA Library Prep Kit for Illumina (NEB) was used to construct the libraries from IP and input RNA. Sequencing was performed on the Illumina Hiseq 2500 platform.

### ChIP‐Qpcr

The relative amounts of Input and IP DNA of all samples were determined using a spectrophotometer (NanoDrop, ND1000). The diluted ChIP DNA was analyzed by qPCR according to the procedure described above for RT–qPCR. qPCR was conducted using SYBR Green qPCR mixture in an Applied Biosystems. Primers used for ChIP‐qPCR are listed in Table S4.

### Analysis of m5C‐RIP‐seq Data and Validation

RIP‐seq data were analyzed according to the procedure previously described.^[^ ^5^ ^]^ Raw sequence quality was evaluated using FastQC, the adapter sequences and low‐quality reads were removed, and then clean reads were aligned using the BWA with MEN algorithm^[^ ^74^ ^]^ to the TAIR 10 genome assembly. MACS2^[^ ^75^ ^]^ was used for peak detection and peaks were considered if their *p* value < 0.00001 and fold change > 2. Three independent RIP‐seq libraries were prepared for WT and *emf1*, the heatmap of correlations revealed high reproducibility between these replicates, and the high‐quality replicates were merged for subsequent analysis. The peaks were annotated according to the genome annotation file using bedtools software, and signal levels was determined by FPKM (IP/input) for the regions of genes and the up‐/down‐stream (2 kb) and visualized using deeptools software. For m^5^C genes validation, RNA was reverse transcribed using a GoScript Reverse Transcription System (Promega). qPCR was conducted using SYBR Green qPCR mixture in an Applied Biosystems. Primers used for RIP‐qPCR are listed in Table S4.

### Gene Ontology Analysis

Gene ontology (GO) enrichment analysis were implemented using the DAVID database,^[^ ^76^ ^]^ GO terms were classified into three categories (biological process, cellular component, and molecular function), these terms with a cutoff (*p* value < 0.05) were considered as the significantly enriched, and visualized in R software.

### Correlation Analysis between Epigenome and Transcription

m^5^C‐modified genes in both WT and *emf1* were divided into three categories (down‐regulated, no change, and up‐regulated) according to the fold change of *emf1*/WT. These gene expression levels in WT and *emf1* were also compared using a log2(FPKM+1) normalized value. There are four categories defined (genes with 1, 2, 3, and >3 peaks) according to m^5^C peaks distribution in the gene body and also divided into two gene groups with high expression level (FPKM ≥ 1) and low expression level (FPKM < 1). m^5^C‐modified photosynthesis, flower, and seed genes were selected to the compare expression level in WT and *emf1*.

### Integrated Analysis of Histone ChIP‐seq/DNA‐IP‐seq and m5C‐IP‐seq Data

Since changes in some histone and DNA modification in WT are not obvious, only four modifications (6mA, 5mC, H3K4me3, and H2AK121ub) were selected for further dynamic analysis and raw data of H3K4me3, 5mC, 6 mA, and H2AK121ub were downloaded from Plant Chromatin State Database (http://systemsbiology.cau.edu.cn/).^[^ ^77^ ^]^ Raw data were re‐analyzedto obtain the significant enrichment regions of these markers and calculated density with 50‐kb windows to profile distribution between these markers and m5C at a genome‐wide scale. The distribution of m^5^C and H3K27me3 was also performed and visualized using a Circos plot.

### Statistics Analysis

For sequencing experiments, *p* values were corrected for multiple testing by false discovery rate (FDR). The statistical significances were determined by FDR‐adjusted *p* value cutoff of 0.00001, except for differential enrichment analyses (between genotypes or treatments) that used 0.05 as a cutoff. For all other statistical comparisons in this study, *p* values were calculated using two‐sided t‐test (equal variance).

## Conflict of Interest

The authors declare no conflict of interest.

## Author Contributions

D.Z., W.G., and T.W. contributed equally to this work. L.P. conceived and supervised the project. D.Z., T.W., F.X., Y.W., and H.W. performed the experiments. W.G., Y.W., and L.L. analyzed the data. L.P., D.Z., and Z. S. wrote the manuscript.

## Supporting information

Supporting Information

Supplemental Table 1

## Data Availability

The data that support the findings of this study are available from the corresponding author upon reasonable request.
