# Supplementary material for: RNA 5‐Methylcytosine Modification Regulates Vegetative Development Associated with H3K27 Trimethylation in Arabidopsis
Source: Adv Sci (Weinh). 2022 Nov 16;10(1):2204885. doi: 10.1002/advs.202204885 (PMC9811455; doi:10.1002/advs.202204885)
Supplement: Supplementary file 1 — Supporting Information [file ADVS-10-2204885-s002.pdf]

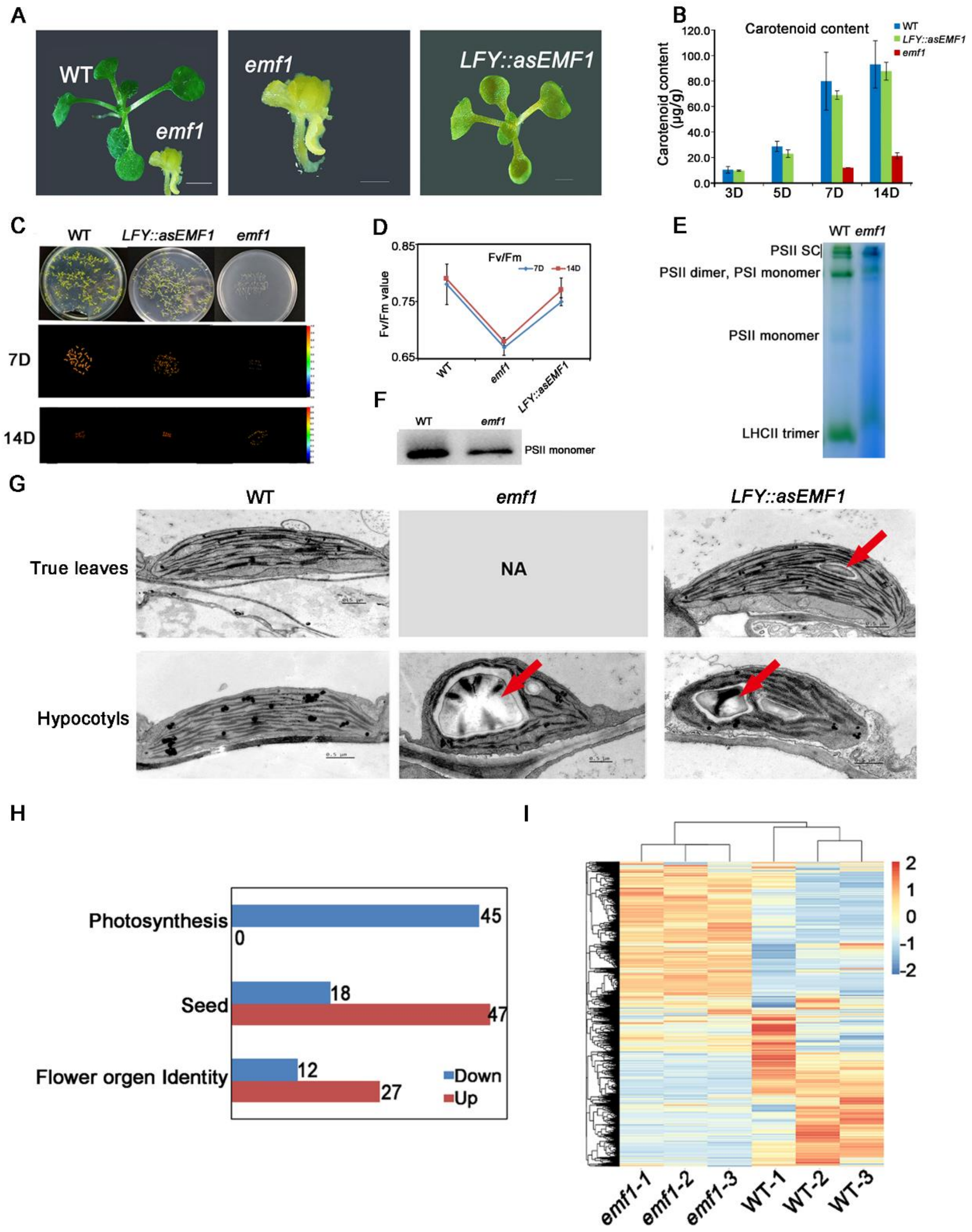

Supplementary Figure 1. Phenotypes of defective photosynthesis in *LFY::asEMF1* and *emf1* seedlings and differentially expressed genes in *emf1* seedlings.

A. Phenotypes of 14-old-day WT, *emf1* and *LFY::asEMF1* plants. BAR= 1mm.

B. Change in carotenoid content of plants in 3-day-old, 5-day-old, 7-day-old, 14-day-old WT, *emf1* and *LFY::asEMF1* seedlings.

C. Chlorophyll fluorescence image of WT, *LFY::asEMF1* and *emf1* seedlings.

D. Analysis of the maximum quantum efficiency of PSII (Fv/Fm) in WT, *emf1* and *LFY::asEMF1* seedlings. Error bars represent ±SD (n=3).

E. Blue Native (BN)-PAGE analysis of thylakoidal protein complexes from WT and *emf1* plants. An 8μg portion of chlorophyll was loaded in each well.

F. Western analysis of thylakoid protein in WT and *emf1* plants. The protein was immunoblotted with D1 antisera. 10-μg portion was loaded on each well.

G. Ultrastructure of chloroplasts of true leaves and hypocotyls from WT, *emf1* and *LFY::asEMF1* under light growth condition. The true leaves are not available in *emf1* mutants. The red arrows indicate starch granules. BAR=0.5μm.

H. Number of differentially expressed photosynthesis, seed and flower organ identity genes in *emf1* seedling.

I. Heatmap of differentially expressed genes in WT and *emf1* seedling revealed by RNA-seq.

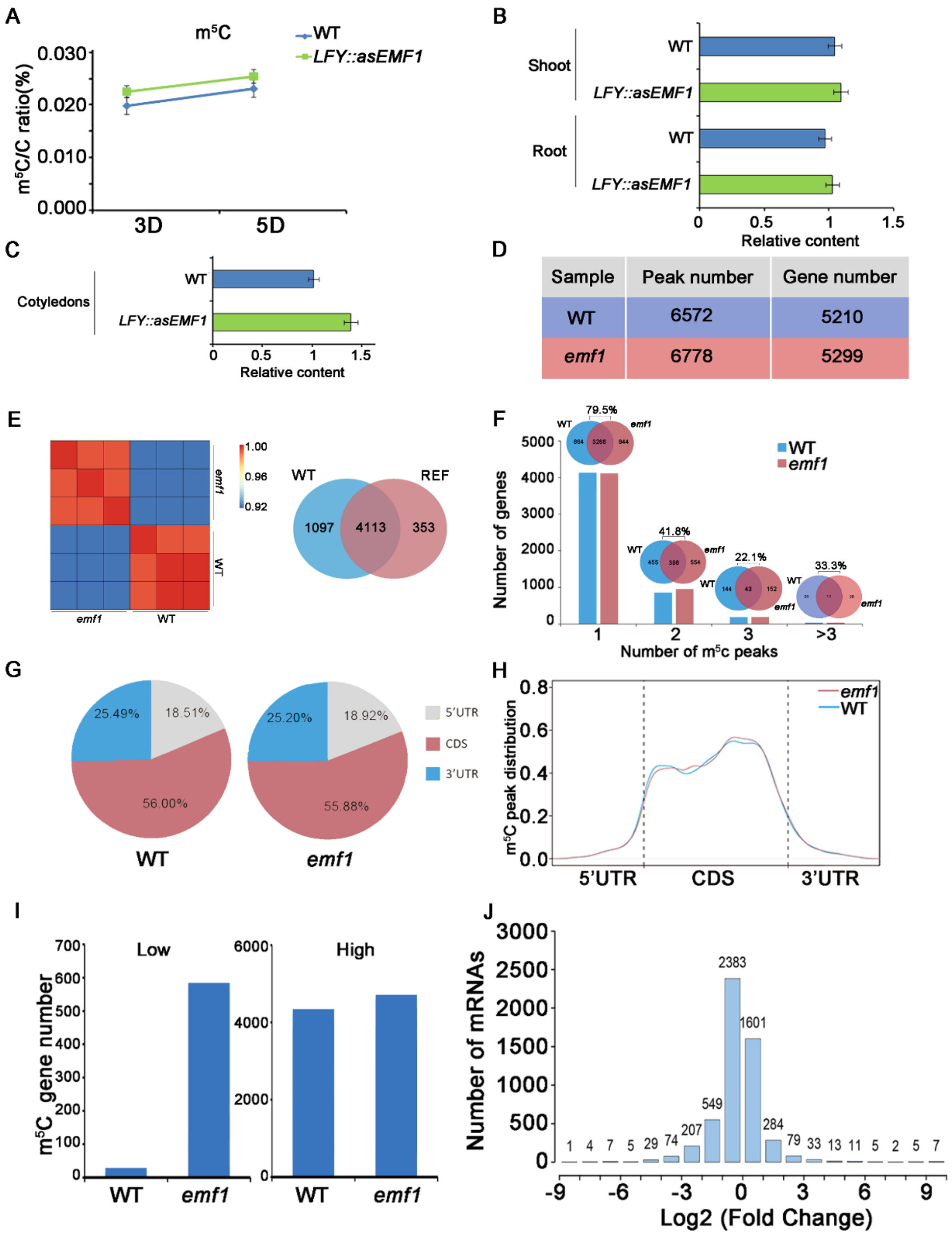

Supplementary Figure 2. Widespread m^{5}C methylation in WT and *emf1* plants.

A. LC-MS/MS assays show quantification of m^{5}C levels in RNA of 3-day-old and 5-day-old WT and *LFY::asEMF1* seedlings.

B and C. LC-MS/MS assays show quantification of m^{5}C levels in RNA from various tissues of WT and *LFY::asEMF1*, including 1 week seedlings Shoot and Root, Cotyledons.

D. Number of m^{5}C peaks and corresponding genes in WT and *emf1* plants by m^{5}C-RIP-seq analysis.

E. Pearson correlation coefficients among normalized signals profiles generated by the m^{5}C-RIP-seq analysis of three biological replicates (left panel). Venn diagram showing overlap between m^{5}C marked transcripts identified via RIP-seq of WT seedlings from our dataset (WT) and published dataset REF[1] (right panel).

F. List of the numbers of genes bearing 1, 2, 3 or >3 m^{5}C peaks in WT and *emf1* seedlings and overlap between m^{5}C marked genes at different m^{5}C methylation site in WT and *emf1* seedlings.

G. Distribution of m^{5}C peaks within protein-coding gene bodies divided into 5'UTRs, CDSs, 3'UTRs in WT and *emf1* are shown in left and right panel, respectively.

H. Distribution pattern of m^{5}C peaks in transcript segments divided into 5' UTRs, CDSs, and 3' UTRs.

I. Comparison of number of m^{5}C methylation genes bearing in WT and *emf1* with high (FPKM≥1) and low (FPKM<1) expression levels. The association between gene number and expression was examined by chi-square test. $p < 10^{-5}$ was considered to be significant.

J. Comparison of number of m^{5}C methylation and regulated genes in *emf1* revealed by RIP-seq and RNA-seq. <-1, down-regulated genes in *emf1*. >1, up-regulated genes in *emf1*. Change of expression genes in *emf1* is p<0.05.

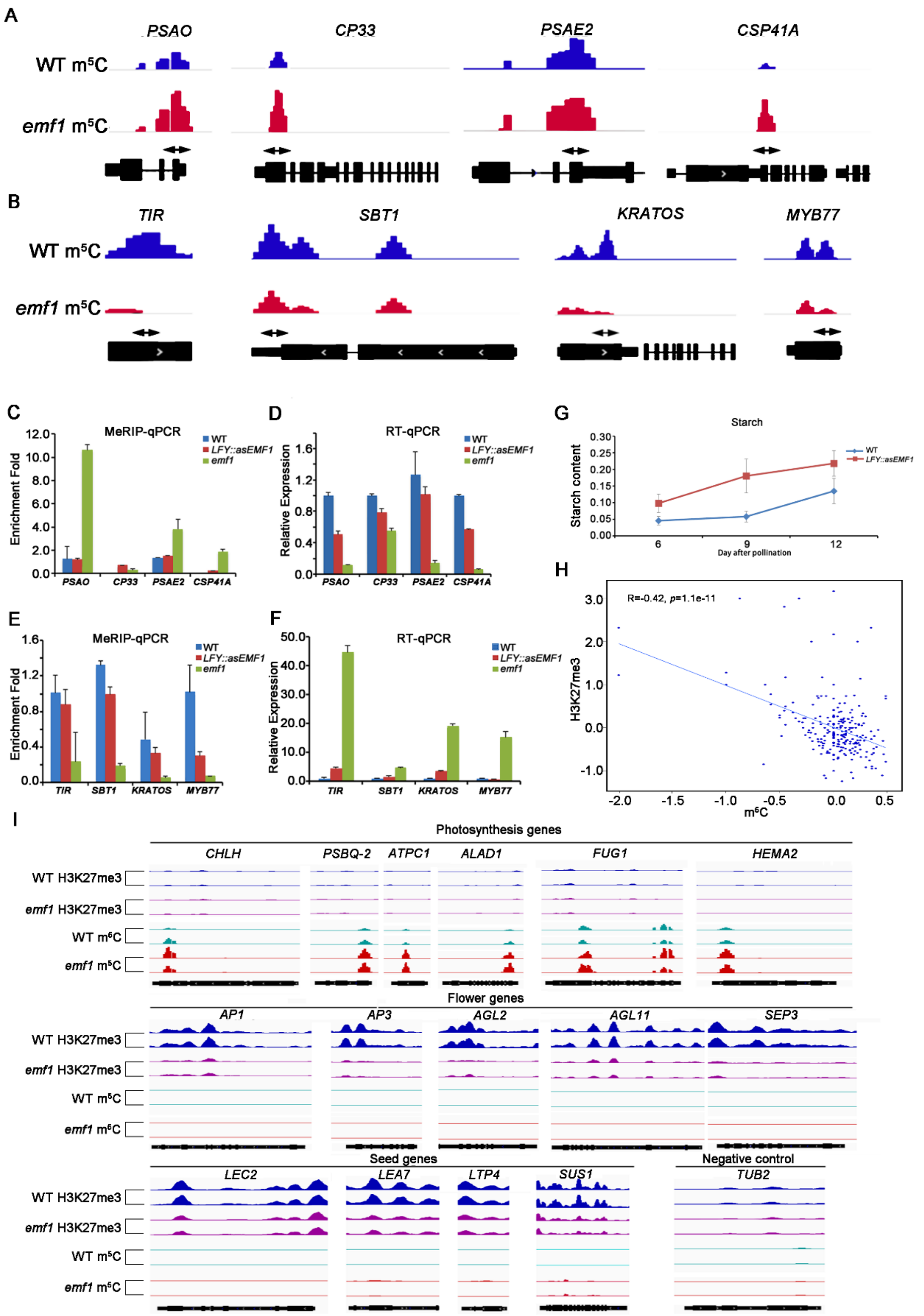

Supplementary Figure 3. m^{5}C is associated with negative gene expression in *emf1* mutants and methylation of EMF1-dependent m^{5}C and H3K27me3 sites in WT and *emf1* plants.

A and B. RIP-seq analysis of m^{5}C distribution at down-regulated genes (A) and up-regulated genes (B) in *emf1* revealed by RNA-seq.

C. RIP-qPCR analysis of m^{5}C levels at the *PSAO*, *CP33*, *PSAE2* and *CSP41A* loci in WT and *emf1* seedlings. Primers (double arrowheads) correspond to the gene regions shown in (A). Quantities of RNA fragments after RIP

were quantified by real-time quantitative PCR (RT-qPCR), and were subsequently normalized to the internal control (*TUB2*).

D. mRNA expression levels of the up-regulated genes *PSAO*, *CP33*, *PSAE2* and *CSP41A* in *emf1* revealed by RIP-seq.

E. RIP-qPCR analysis of m^5C levels at the *TIR*, *SBT1*, *KRATOS* and *MYB77* loci in WT, *emf1* plants seedlings. Primers (double arrowheads) correspond to the gene regions shown in (B). Quantities of RNA fragments after RIP were quantified by RT-qPCR, and were subsequently normalized to the internal control (*TUB2*).

F. mRNA expression levels of the down-regulated genes *TIR*, *SBT1*, *KRATOS* and *MYB77* in *emf1* revealed by RIP-seq.

G. Changes of starch content of seeds in WT, *LFY::asEMF1* plans during seed development. Values are mean ± SD of measurements on three biological replicates.

H. Pearson correlation coefficient analysis of m^5C signals with H3K27me3.

I. ChIP-seq and RIP-seq analysis of H3K27me3 and m^5C distribution at Photosynthesis genes, Flower genes, Seed genes and negative control gene (*TUB2*).

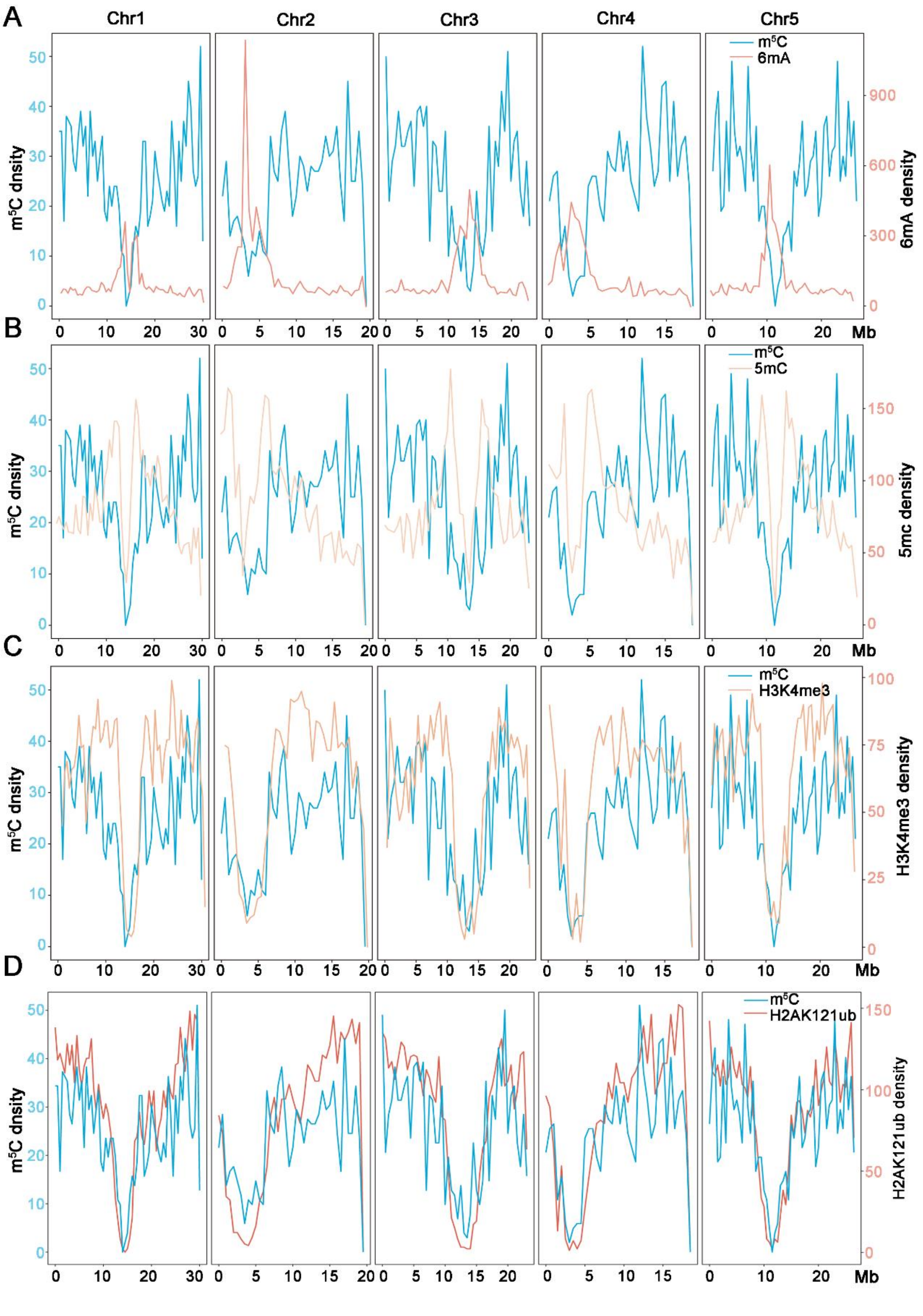

Supplementary Figure 4. Distribution of m^5C and other Histone/DNA modifications along five *Arabidopsis* chromosomes.

A. Distribution of m^5C (blue) and 6mA (orange) along five *Arabidopsis* chromosomes.

B. Distribution of m^5C (blue) and 5mC (orange) along five *Arabidopsis* chromosomes.

C. Distribution of m^5C (blue) and H3K4me3 (orange) along five *Arabidopsis* chromosomes.

D. Distribution of m^5C (blue) and H2AK121ub (orange) along five *Arabidopsis* chromosomes.

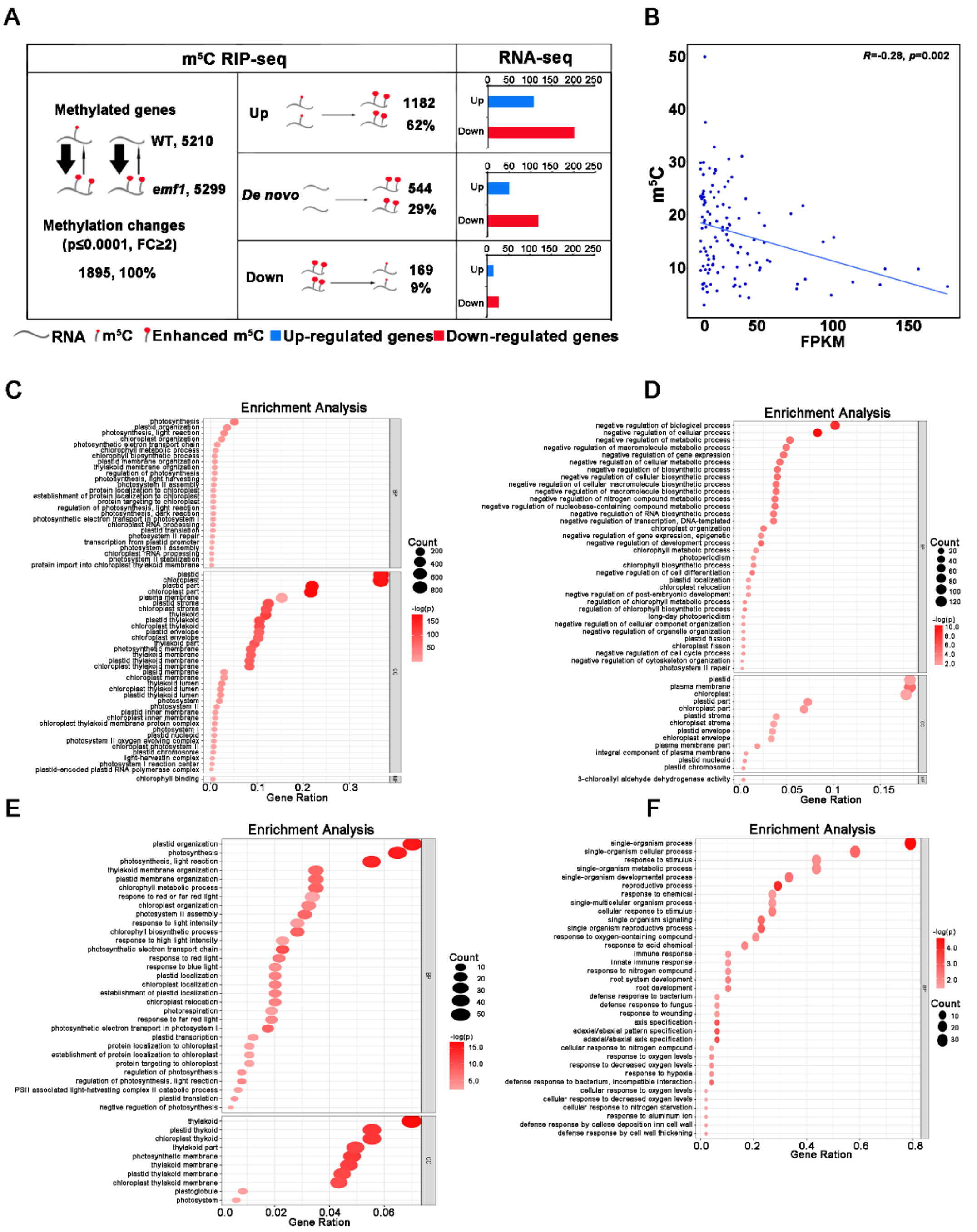

Supplementary Figure 5. m^{5}C modified genes and expression in *emf1* mutants.

A. Number of up- or down-regulated genes in *emf1* mutants containing m^{5}C modification revealed by m^{5}C-RIP-seq and RNA-seq.

B. Pearson correlation coefficient analysis of m^{5}C signals with FPKM in *emf1* mutants.

C. Gene Ontology (GO) enrichment analysis of genes without H3K27me3 modifications. BP, biological process. CC, cell component. MF, molecular function. Statistical analysis was performed using the DAVID tool. p<0.05.

D, E and F. GO enrichment analysis of genes bearing m^{5}C modifications. up-regulated genes in *emf1* (D), *de novo* genes in *emf1* (E), down-regulated genes in *emf1* (F). BP, biological process. CC, cell component. MF, molecular function.

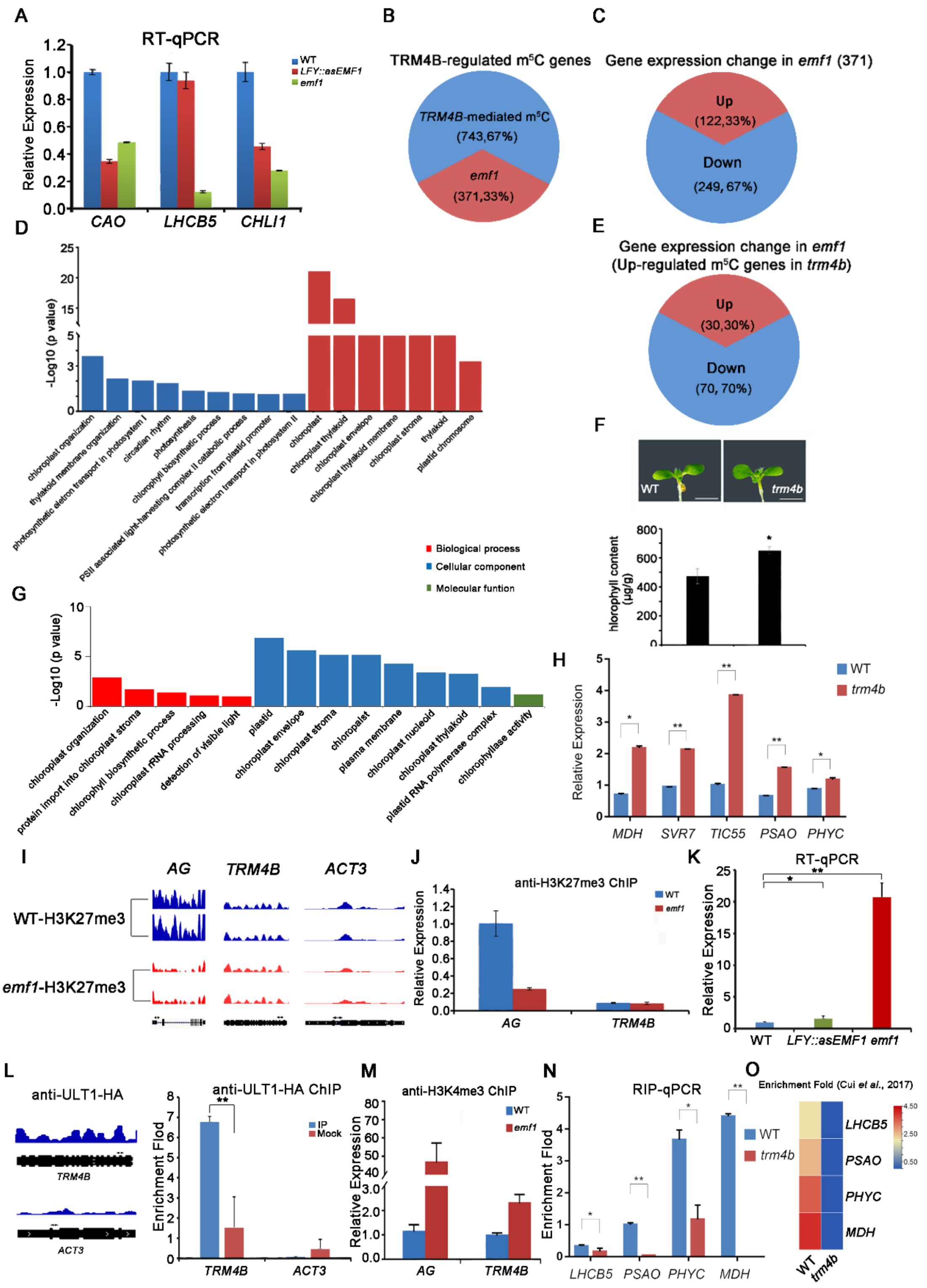

Supplementary Figure 6. Gene expression changes in *emf1* seedlings and *EMF1* affects histone methylation at m^{5}C methyltransferase gene *TRM4B*.

A. RT-qPCR analyses of *LHCB5*, *CAO*, *CHLI1* expression in WT, *LFY::asEMF1* and *emf1* seedlings. Gene expression levels in WT seedlings are set as 1. Error bars, mean±SD.

B. Overlap between TRM4B-regulated m^{5}C genes and EMF1-regulated genes in *emf1*.

C. Gene with expression change for TRM4B-regulated m^{5}C genes and EMF1-regulated genes in *emf1*. Up,

up-regulated genes in *emf1*. Down, down-regulated genes in *emf1*.

D. GO enrichment analysis biological process (blue) and cell component (red) of down-regulated genes in *emf1* mutants. Statistical analysis was performed using the DAVID tool. p <0.05.

E. Gene with expression change for up-regulated m^5C genes in *trm4b* and EMF1-regulated genes in *emf1*. Up, up-regulated genes in *emf1*. Down, down-regulated genes in *emf1*.

F. Phenotypes of 7-old-day WT and *trm4b* plants. BAR= 5mm. Chlorophyll content in WT and *trm4b* seedlings. Error bars represent ±SD (n=6).

G. GO enrichment analysis of TRM4B-regulated m^5C genes with up-regulated expression in *trm4b*.

H. RT-qPCR analyses of *MDH, SVR7, TIC-55, PSAO, PHYC* expression in WT and *trm4b* seedlings. Gene expression levels in WT seedlings are set as 1. Error bars, mean±SD.

I. ChIP-seq analysis of H3K27me3 distribution at *AG*, *TRM4B* and negative control gene (*ACT3*). The white arrowheads indicate transcriptional direction. The double arrowheads above each gene model indicate the regions amplified for the ChIP–qPCR analysis in (J), (L) and (M).

J. ChIP-qPCR analysis of H3K27me3 levels at the TRM4B locus in WT and *emf1* mutant plants. Quantities of DNA fragments after ChIP were quantified by qPCR, and were subsequently normalized to the internal control (*ACT3*). The flower MADS box gene *AGAMOUS* (*AG*) was used as a positive control of H3K27me3-marked gene.

K. RT-qPCR analyses of *ULT1* expression in WT, *LFY::asEMF1* and *emf1* seedlings. Gene expression levels in WT seedlings are set as 1. Error bars, mean±SD.

L. ChIP-seq and ChIP-qPCR analysis of ULT1 binding at *TRM4B* and *ACT3*. ChIP-seq analysis of ULT1 binding distribution at the *TRM4B* gene locus from our previous report[2] (left panel). The graphs show ChIP–qPCR using an anti-HA antibody (IP) or no antibody (mock control) as a percentage of input DNA, with error bars indicating SD (right panel).

M. ChIP-qPCR analysis of H3K4me3 levels at the *TRM4B* loci in WT and *emf1*. Quantities of DNA fragments after ChIP were quantified by qPCR, and were subsequently normalized to the internal control (*ACT3*). The flower MADS box gene *AG* was used as a positive control of H3K4me3-marked gene.

N. m^5C-IP-qPCR analysis of m^5C modification on mRNA of *LHCB5, PSAO, PHYC, MDH* in WT and *trm4b* seedlings.

O. m^5C Enrichment Fold of photosynthesis- and chloroplast- related genes in WT and *trm4b* seedlings from published dataset[1].

A

	m^{5}C gene in *emf1* (5299)	TRM4B-dependent m^{5}C gene (3362)
EMF1-no-H3K27me3 gene (2219)	775	458
%	34.9	59.1

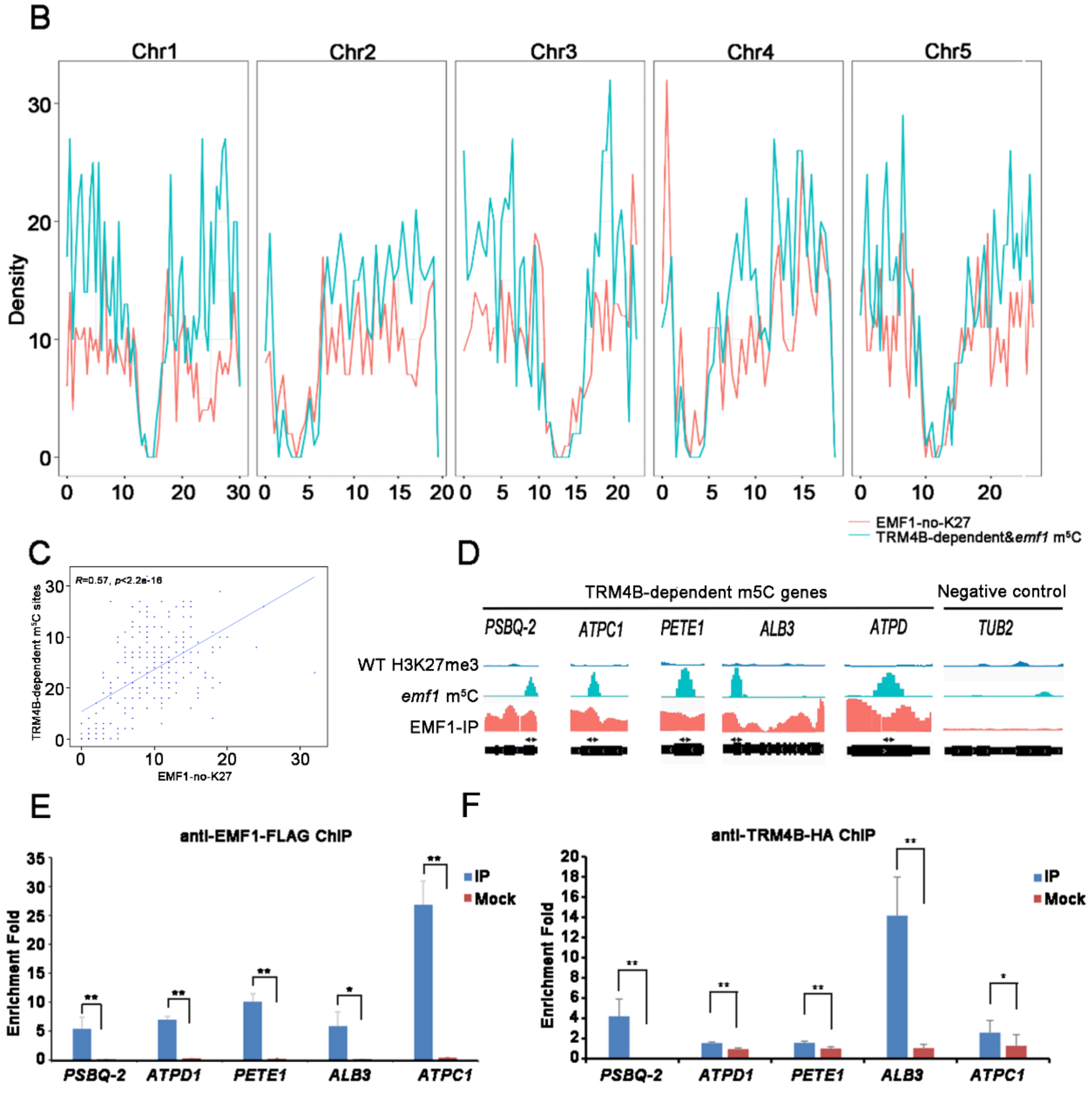

Supplementary Figure 7. Analysis of TRM4B-dependent m^{5}C and EMF1-no-H3K27me3 genes.

A. Analysis of m^{5}C gene and TRM4B-dependet m^{5}C gene in EMF1-no-H3K27me3.

B. Distribution of EMF1-no-H3K27me3 (EMF1-no-K27) and TRM4B-dependet m^{5}C gene in *emf1* seedling along five *Arabidopsis* chromosomes.

C. Pearson correlation coefficient analysis of TRM4B-dependent m^{5}C sites with EMF1-no-H3K27me3 (EMF1-no-K27).

D. ChIP-seq and RIP-seq analysis of H3K27me3 and m^{5}C distribution at TRM4B-dependent m^{5}C gene and negative control genes (*TUB2*).

E. ChIP-qPCR analysis of EMF1 binding at *PSBQ-2*, *ATPD1*, *PETE1*, *ALB3* and *ATPC1*. The graphs show ChIP–qPCR using an anti-FLAG antibody (IP) or no antibody (mock control) as a percentage of input DNA, with error bars indicating SD.

F. ChIP-qPCR analysis of TRM4B binding at *PSBQ-2*, *ATPD1*, *PETE1*, *ALB3* and *ATPC1*. The graphs show ChIP–qPCR using an anti-HA antibody (IP) or no antibody (mock control) as a percentage of input DNA, with error bars indicating SD.

Reference

[1] X. Cui, Z. Liang, L. Shen, Q. Zhang, S. Bao, Y. Geng, B. Zhang, V. Leo, L. A. Vardy, T. Lu, X. Gu,H. Yu, *Mol.Plant* **2017,** *10,* 1387-1399.

[2] F. Xu, T. Kuo, Y. Rosli, M. S. Liu, L. Wu, L. O. Chen, J. C. Fletcher, Z. R. Sung,L. Pu, *Mol.Plant* **2018,** *11*, 659-677.
